# Association between obesity indices, insulin resistance markers, and osteoarthritis in middle-aged and elderly Chinese adults

**DOI:** 10.3389/fnut.2025.1627421

**Published:** 2025-10-31

**Authors:** Suyao Zhang, Zhen Jiang, Huayuan Liao, Huwei Bian, Junan Zhou, Haibo Wang, Tao Jiang

**Affiliations:** Changzhou Hospital Affiliated to Nanjing University of Chinese Medicine, Changzhou, Jiangsu, China

**Keywords:** obesity indices, insulin resistance surrogates, osteoarthritis, cross-sectional study, middle-aged and older adults

## Abstract

**Background:**

Previous studies have indicated an association between osteoarthritis (OA), obesity, and insulin resistance (IR). However, current literature lacks sufficient clinical data to fully elucidate the relationship between obesity indices, insulin resistance surrogates (IR surrogates), and OA in China's middle-aged and elderly population. This study aims to investigate the correlation between obesity indices [body fat percentage (BFP), lipid accumulation product (LAP), body mass index (BMI), waist-to-height ratio (WHtR)], IR surrogates [triglyceride-glucose (TyG) index and its derivatives: TyG with waist circumference (TyG-WC), TyG-BMI, TyG-WHtR, and OA risk, and evaluate the diagnostic efficacy of these indices for OA.

**Methods:**

This study utilized data from the China Health and Retirement Longitudinal Study (CHARLS). Multivariable logistic regression and Cox proportional hazards models were employed, alongside Receiver Operating Characteristic (ROC) curves, restricted cubic splines, and subgroup analyses, to assess the associations between obesity indicators, IR surrogates, and the risk of OA in middle-aged and older adults.

**Results:**

A multivariable logistic regression analysis was conducted using data from 10,457 participants, of whom 3,667 were diagnosed with OA. In fully adjusted models, all indices as continuous variables were positively associated with OA risk (all *p* < 0.05): BFP (95% CI: 1.02–1.04), LAP (95% CI: 1.04–1.15), BMI (95% CI: 1.02–1.05), WHtR (95% CI: 1.10–1.21), TyG (95% CI: 1.02–1.20), TyG-WC (95% CI: 1.06–1.18), TyG-BMI (95% CI: 1.10–1.22), and TyG-WHtR (95% CI: 1.14–1.32). ROC analysis indicated TyG-WHtR had the greatest predictive ability for OA risk (AUC = 0.680). A multivariable Cox regression analysis of TyG-WHtR in 5,718 participants, among whom 1,827 developed OA during a median follow-up of 108 months, showed each one-unit increase in TyG-WHtR was associated with a 20% higher risk of OA (95% CI: 1.11–1.31). Trend tests revealed a significant dose–response relationship (*p* < 0.05).

**Conclusion:**

Obesity-related indicators and IR surrogates are significantly associated with OA risk. Among these, TyG-WHtR demonstrates the strongest predictive performance, suggesting its potential as an early screening tool for OA. This study highlights obesity and IR as modifiable risk factors, providing a basis for the early prevention and control of OA.

## Introduction

Osteoarthritis (OA) is a common degenerative joint disorder, characterized primarily by the progressive degradation of articular cartilage, synovial inflammation, and osteophyte formation. It typically manifests as joint pain, stiffness, swelling, and limited range of motion (1, 2). OA significantly impairs patients' quality of life while increasing healthcare resource utilization and disability-related costs, imposing a substantial economic burden (3, 4). According to recent epidemiological studies, approximately 595 million people (7.6% of the global population) had OA in 2020, with projections suggesting this number will reach 1 billion by 2050. Notably, the prevalence of knee, hand, and hip OA is expected to increase by 74.9%, 48.6%, and 78.6%, respectively, whereas other forms of OA may rise by 95.1% (5). National studies further indicate that since 1990, OA incidence and mortality rates in China have risen annually, exceeding the global average (6). These findings underscore the significant challenges China faces in OA prevention and management.

OA is a complex condition involving the entire joint and affecting multiple tissues (7, 8). Its risk factors include sex, mechanical loading, chronic inflammation, and genetic susceptibility, reflecting the interplay of multiple intra- and extra-articular tissues and mechanisms (9, 10). In knee OA, cartilage degeneration is considered the central pathological feature, characterized by degradation of the cartilage matrix, disruption of collagen fibers, and loss of proteoglycans (11). These changes lead to impaired shock absorption and load-bearing capacity of the cartilage (12). Additionally, bone remodeling and osteophyte formation are also prominent features of OA (13). Accelerated subchondral bone remodeling can be observed even in early OA, manifesting as increased bone resorption and aberrant bone formation, resulting in trabecular thinning, bone marrow lesions, and micro-fractures (14).

Beyond cartilage and bone abnormalities, tendons and ligaments in OA patients often exhibit tissue degeneration, reduced mechanical strength, and infiltration of inflammatory cells, which contribute to joint instability and exacerbate cartilage damage (15). Low-grade synovitis has also been recognized as a key characteristic of OA, clinically presenting as mild synovial hyperplasia, angiogenesis, and inflammatory cell infiltration (16). This process not only aggravates cartilage degradation but is also strongly associated with pain (17).

In the pathogenesis of OA, the infrapatellar fat pad (IFP) plays a critical role as an important soft tissue structure (18). On one hand, the IFP exerts protective effects through the secretion of anti-inflammatory factors and provision of stem cells; on the other hand, under pathological conditions, it becomes a driver of inflammation and fibrosis (19, 20). The biological properties of the IFP allow it to act as a reservoir of inflammatory factors, and its high susceptibility to change can disrupt the stability of surrounding tissues (21). Moreover, obesity exacerb OA progression by altering IFP function (22). Studies using selective lipodystrophy mouse models suggest that systemic metabolic and inflammatory effects play a major role in disturbing cartilage homeostasis, with intra-articular adipose tissue serving a regulatory function in this process (22).

However, recent studies have shown that metabolic factors, particularly obesity and insulin resistance (IR), also play significant roles in the onset and progression of OA (23). IR refers to the impaired ability of insulin to effectively promote glucose uptake and utilization, resulting in glucose metabolism abnormalities (24). Growing evidence indicates a strong positive correlation between IR and OA (25–27). Adipose tissue, as an active endocrine organ, plays a significant role in the onset and progression of OA through the secretion of various adipokines and inflammatory mediators (28). Studies have shown that obesity-induced adipose tissue dysfunction can lead to increased secretion of pro-inflammatory adipokines (such as leptin and resistin) and decreased secretion of anti-inflammatory adipokines (such as adiponectin), thereby creating a systemic and local inflammatory environment conducive to the development of OA (29). Furthermore, insulin resistance promotes the progression of OA through multiple pathways (30). A state of hyperglycemia can lead to the accumulation of advanced glycation end products, which bind to receptors on the surface of chondrocytes, triggering oxidative stress and inflammatory responses that accelerate the degradation of the cartilage matrix (31). Adipose tissue dysfunction is closely associated with metabolic syndrome and influences OA-related pain through various pain mechanisms, including nociceptive pain, peripheral sensitization, and central sensitization (32).

Although traditional diagnostic methods, such as the hyperinsulinemic-euglycemic clamp technique, are considered the gold standard for assessing IR, their invasiveness and high cost limit their application in large-scale epidemiological studies (33). To address these limitations, alternative markers based on blood glucose and lipid profiles, such as the triglyceride-glucose index (TyG), have been widely used to evaluate IR and are closely associated with the prevalence of metabolic diseases, including cardiovascular disease, ischemic stroke, and diabetes (34–36). As a major risk factor for OA, obesity interacts with IR to exacerbate OA progression (37). Recent research has proposed combining TyG with various obesity indicators to create novel indices, such as TyG-waist circumference (TyG-WC), TyG-body mass index (TyG-BMI), and TyG-waist-to-height ratio (TyG-WHtR). These emerging metabolic markers have demonstrated high accuracy and sensitivity in assessing metabolic disease risk and show potential for application in OA risk assessment (38).

Although previous studies have examined the relationship between obesity indicators, IR surrogates, and OA, systematic research specifically addressing the association between IR surrogates and OA remains limited, particularly in the Chinese population, especially among middle-aged and elderly individuals. Given the high prevalence of OA in this demographic, the aim of this study is to investigate the associations between various obesity indicators and IR surrogates, including body fat percentage (BFP), lipid accumulation product (LAP), body mass index (BMI), waist-to-height ratio (WHtR), TyG, TyG-WC, TyG-BMI, and TyG-WHtR, and OA risk. Furthermore, this study seeks to evaluate the potential utility of these indicators in the early screening and intervention of OA, thereby providing new theoretical insights and research directions for its prevention and treatment.

## Materials and methods

### Data source

The data for this study were derived from the China Health and Retirement Longitudinal Survey (CHARLS), a project administered by the China Social Science Survey Center at Peking University. This survey aims to provide comprehensive data on the health, economic status, and social behaviors of middle-aged and older adults in China, with a particular focus on the challenges of population aging. The national baseline survey of CHARLS (CHARLS 2011) was conducted from June 2011 to March 2012 using a multi-stage probability sampling method. The survey covered 150 counties (districts) and 450 villages/community committees, enrolling 17,708 participants. Since the baseline survey, CHARLS has conducted biennial, face-to-face, computer-assisted personal interviews to collect demographic, socioeconomic, biomedical, health, and functional data from this population. Additionally, blood samples were collected from participants in the 2011 survey, yielding a rich source of biomarker data for subsequent analyses.

This study utilizes data from the CHARLS, collected between 2011 and 2020. A combination of cross-sectional and longitudinal cohort designs was employed. The cross-sectional analysis incorporates data from participants surveyed in the 2011–2012 wave of CHARLS, as well as those newly enrolled in the 2015–2016 wave. The longitudinal cohort was established with participants from the 2011 baseline (Wave 1) and followed through subsequent waves. The dataset comprises comprehensive information on biological markers, physical examination data, and responses to structured questionnaires, covering participants' general health status, lifestyle factors, and history of chronic diseases. All data were collected by trained surveyors using standardized methods to ensure high quality and reliability. All participants provided written informed consent, and the study protocol was approved by the Biomedical Ethics Committee of Peking University.

### Exclusion criteria

The exclusion criteria were as follows: (1) adults under 45 years of age and those with missing age data; (2) participants with missing OA outcome data; (3) participants with missing or abnormal data for BFP, LAP, TyG, BMI, WHtR, TyG-WC, TyG-BMI, or TyG-WHtR. For the longitudinal cohort analysis, we applied the following additional exclusion criteria: (1) participants with OA at baseline; (2) those with a follow-up duration of less than 2 years; (3) those with missing OA data during the follow-up period.

### Study variables

#### Exposure and outcome

The primary outcome of this study was the presence of OA, defined as a physician-confirmed diagnosis of OA in the past.

The formulas for calculating BFP, LAP, TyG, BMI, WHtR, TyG-WC, TyG-BMI, and TyG-WHtR are provided in Table 1. Height was measured using a Seca™ 213 stadiometer (Seca Medical Scales and Measuring Systems, Hangzhou, China), and weight was assessed using an Omron™ HN-286 scale (Omron Healthcare, Yangzhou, China). Waist circumference was measured with a flexible tape measure. Blood biomarkers were collected by healthcare professionals from the Chinese Center for Disease Control and Prevention (CDC) following standardized protocols. All participants provided fasting venous blood samples, which were immediately sent to a central laboratory for rigorous analysis to ensure data accuracy and reliability.

**Table 1:** Formulas for obesity indices and insulin resistance surrogates.

**Characteristic**	**Calculation formula**
TyG	Ln (fasting TG [mg/dl] × FBG [mg/dl]/2)
BMI	Weight (kg)/Height ^2^[m]
WHtR	WC (cm)/Height (cm)
TyG-WC	TyG × WC (cm)
TyG-BMI	TyG × BMI (kg/m^2^)
TyG-WHtR	TyG × (WC [cm]/Height [cm])
BFP (Male)	1.20 × BMI + 0.23 × age – 16.2
BFP (Female)	1.20 × BMI + 0.23 × age – 5.4
LAP (Male)	LAP = (WC [cm] – 65) × TG (mmol/L)
LAP (Female)	LAP = (WC [cm] – 58) × TG (mmol/L)

Weight, Body Weight; Height, Body Height; TyG, triglyceride-glucose index; TG, Triglycerides; FBG, fasting blood glucose; BMI, body mass index; WHtR, waist-to-height ratio; WC, waist circumference; BFP, body fat percentage; HDL-C, high-density lipoprotein cholesterol; LAP, lipid accumulation product.

#### Covariate assessment

The covariates in this study comprised: (1) demographic factors (age, sex, marital status, residence place, and education); (2) lifestyle factors (smoke, drink, sleep duration, life satisfaction, and self-rated health); (3) chronic diseases and health conditions, including hypertension, diabetes, dyslipidemia, stroke, cancer, lung diseases, heart diseases, liver diseases, kidney diseases, stomach diseases; (4) biomarkers: high-density lipoprotein (HDL) cholesterol (mg/dl), low-density lipoprotein (LDL) cholesterol (mg/dl), C-reactive protein (mg/L), and uric acid (mg/dl); and (5) health events, specifically falls in the past 2 years.

### Statistical analysis

In this study, continuous variables with a normal distribution were summarized using the mean and standard deviation (SD), whereas those with a non-normal distribution were described using the median and interquartile range (IQR). Categorical variables were expressed as frequencies and percentages. Variables with more than 20% missing data were excluded from the analysis. For the remaining missing data, values were imputed using the random forest method. Prior to correlation analysis, multicollinearity among all predictor variables was assessed using the variance inflation factor (VIF). Variables with a VIF greater than 5 were excluded to mitigate potential multicollinearity issues (Supplementary Table S1).

**Figure 1:**
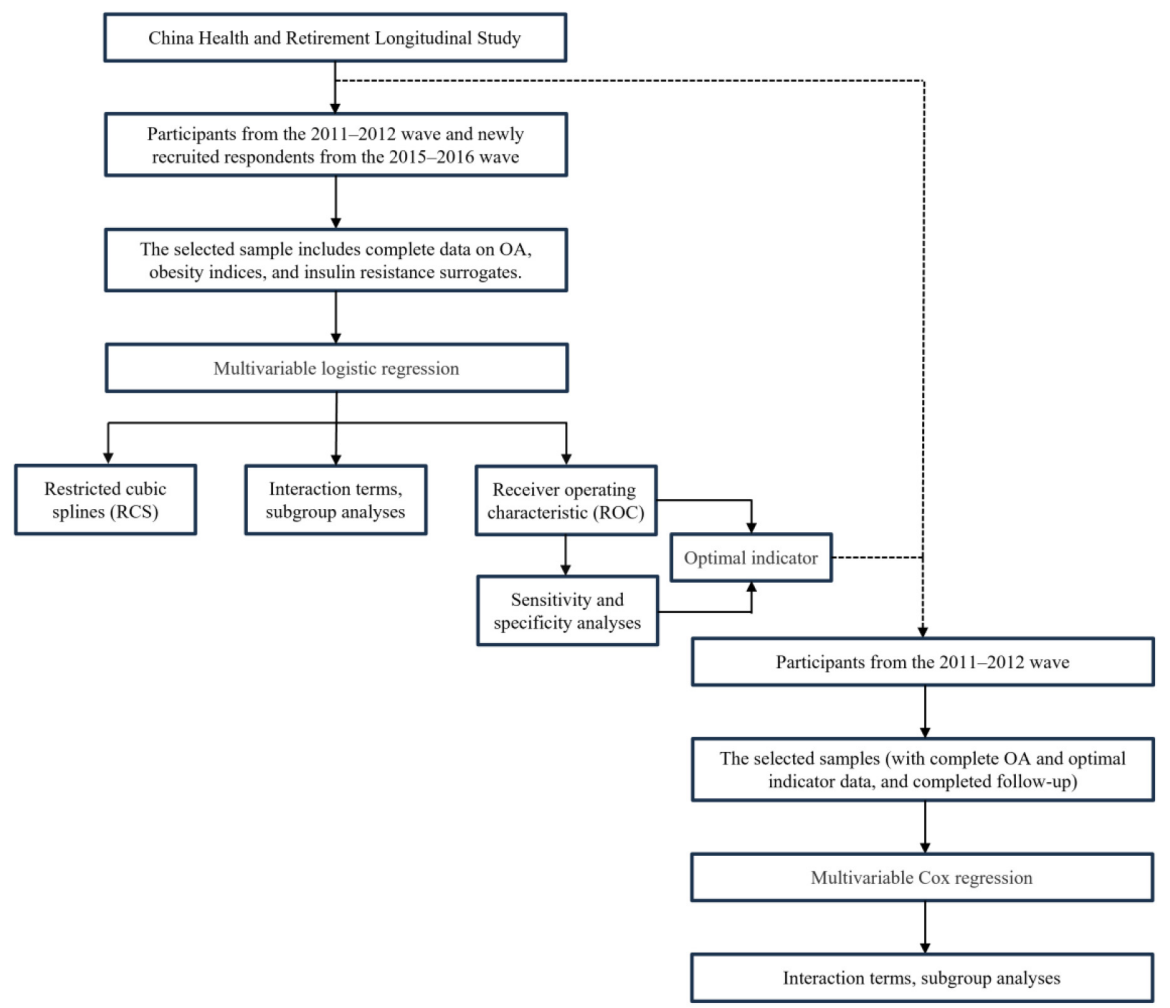
Statistical analysis procedure.

This study consists of two parts (Figure 1):

(1) Cross-sectional analysis: we performed multivariable logistic regression to evaluate the association between obesity indices (BFP, LAP, TyG, BMI, WHtR, TyG-WC, TyG-BMI, and TyG-WHtR) and the risk of OA. Effect estimates were compared across different models to assess the robustness of these indices in relation to OA risk under varying levels of covariate adjustment. We also employed restricted cubic spline (RCS) regression to explore potential non-linear relationships. To examine potential interaction effects, a subgroup analysis was conducted by gender. Finally, receiver operating characteristic (ROC) analysis was used to evaluate the diagnostic performance of each index for OA. Sensitivity and specificity were calculated to validate diagnostic stability and to ensure their reliability and accuracy in clinical settings. All models reported odds ratios (ORs) with corresponding 95% confidence intervals (CIs) and *p*-values.

(2) Longitudinal analysis: we utilized multivariable Cox regression to investigate the most stable and optimal indices for predicting OA risk, as identified in the cross-sectional analysis. To further assess potential interaction effects between this indicator, covariates, and OA, we conducted subgroup analyses stratified by factors including sex, education, marital status, residence place, drink, smoke, hypertension, diabetes, and dyslipidemia. This aimed to identify potential effect modifiers and to evaluate the robustness of the primary findings across different patient subgroups. All models reported hazard ratios (HRs) with corresponding 95% CIs and *p*-values.

In both cross-sectional and longitudinal regression analyses, we progressively adjusted for confounding factors by constructing the following three models:

- Model 1: no covariates adjusted;
- Model 2: adjusted for sex, age, marital status, residence place, and education;
- Model 3: further adjusted for smoke, drink, sleep duration, life satisfaction, self-rated health, hypertension, diabetes, dyslipidemia, stroke, cancer, lung diseases, heart diseases, liver diseases, kidney diseases, stomach diseases, HDL cholesterol (mg/dl), LDL cholesterol (mg/dl), C-reactive protein (mg/L), uric acid (mg/dl), and history of falls in the past 2 years, in addition to the covariates included in Model 2.

Statistical analyses were performed using R version 4.4.3 (https://www.R-project.org/), with statistical significance set at *p* < 0.05.

## Results

### Study population and demographic characteristics

**Figure 2:**
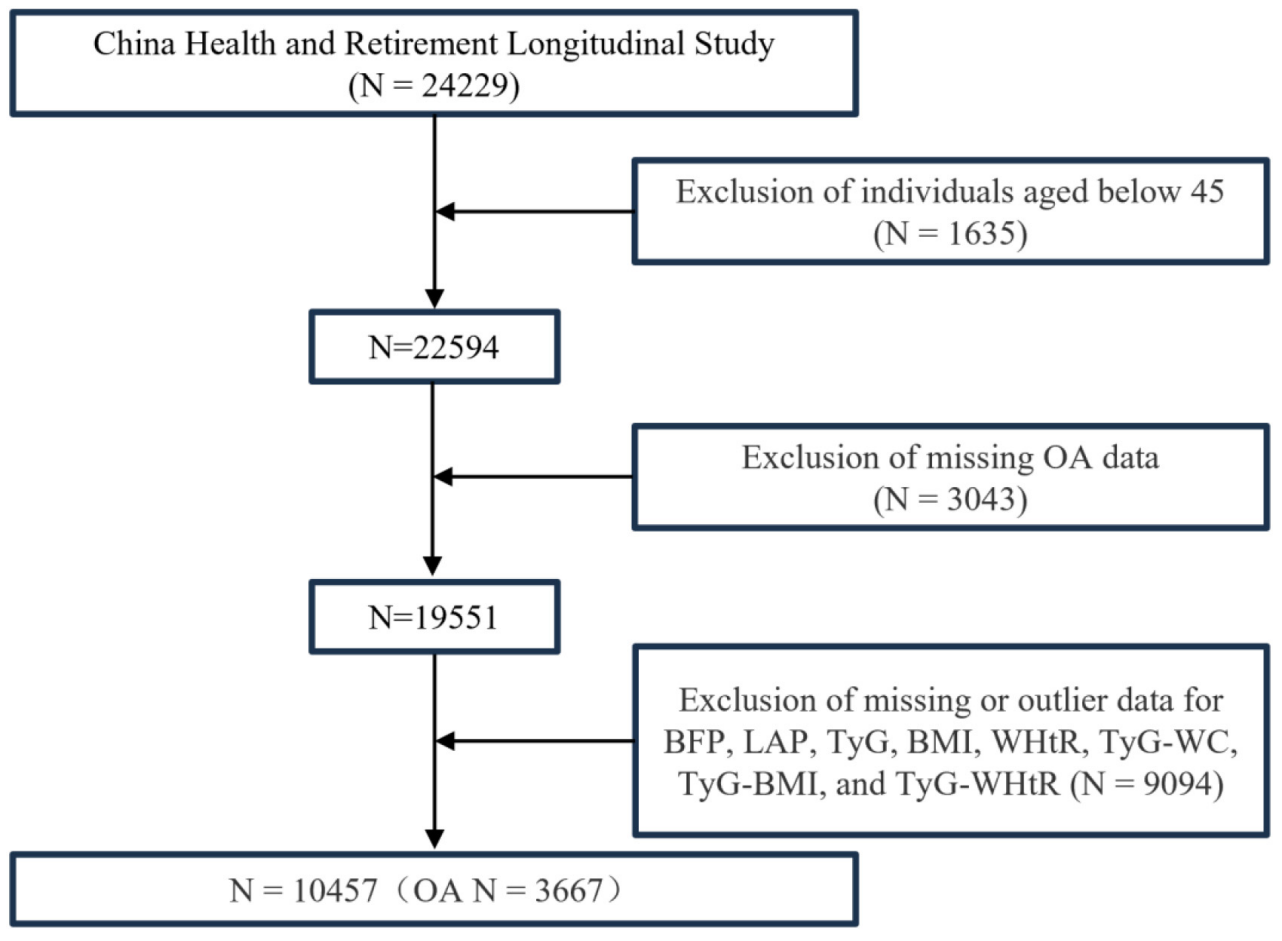
Multivariate logistic regression population screening process.

Data from the CHARLS were analyzed, incorporating participants from the 2011–2012 wave and newly recruited respondents from the 2015–2016 wave (total *N* = 24,229). After excluding participants under 45 years of age and those with incomplete data for OA, BFP, LAP, TyG, BMI, WHtR, TyG-WC, TyG-BMI, and TyG-WHtR, 10,457 individuals were included in the final analysis. Among these, 3,667 (35.07%) were diagnosed with OA (Figure 2).

Demographic analysis revealed that the OA group exhibited characteristics typical of an aging population, with significantly higher proportions of women and rural residents than the non-OA group. The OA group also had a higher percentage of unmarried individuals and lower overall educational attainment. Lifestyle factors significantly differed, with lower rates of smoking and alcohol consumption in the OA group. The prevalence of chronic diseases, including dyslipidemia, stroke, pulmonary disease, heart disease, liver disease, kidney disease, and gastrointestinal disorders, was significantly higher in the OA group. Surveys on life satisfaction indicated that a significantly larger proportion of the OA group reported being “completely dissatisfied” or “somewhat dissatisfied” compared to the non-OA group. Notably, the OA group reported a significantly higher incidence of falls in the preceding 2 years and had a lower median sleep duration. Biomarker analysis showed no significant intergroup differences in CRP, HDL, or LDL levels. The baseline characteristics of the study population are detailed in Table 2.

**Table 2:** Baseline characteristics of the study participants.

**Characteristic**	**OA**			***p*-value**
	**Overall** ***N*** = **10,457**	**No** ***N*** = **6,790**	**Yes** ***N*** = **3,667**	
Age, Median (Q1, Q3)	58 (52, 65)	58 (51, 65)	59 (53, 66)	< 0.001^a^
**Sex**, ***n*** **(%)**				
Female	5,593 (53.5%)	3,409 (50.2%)	2,184 (59.6%)	< 0.0012
Male	4,864 (46.5%)	3,381 (49.8%)	1,483 (40.4%)	
**Education**, ***n*** **(%)**				
Primary school and blow	7,291 (69.8%)	4,493 (66.2%)	2,798 (76.4%)	< 0.001^b^
Junior high school and above	3,158 (30.2%)	2,292 (33.8%)	866 (23.6%)	
**Marital status**, ***n*** **(%)**				
Not married	1,309 (12.5%)	798 (11.8%)	511 (13.9%)	0.001^b^
Married	9,147 (87.5%)	5,992 (88.2%)	3,155 (86.1%)	
**Residence place**, ***n*** **(%)**				
Rural	6,686 (63.9%)	4,230 (62.3%)	2,456 (67.0%)	< 0.001^b^
Urban	3,771 (36.1%)	2,560 (37.7%)	1,211 (33.0%)	
**Drink**, ***n*** **(%)**				
No	6,276 (60.1%)	4,013 (59.2%)	2,263 (61.8%)	0.008^b^
Yes	4,170 (39.9%)	2,771 (40.8%)	1,399 (38.2%)	
**Smoke**, ***n*** **(%)**				
No	6,323 (60.5%)	3,962 (58.4%)	2,361 (64.4%)	< 0.001^b^
Yes	4,132 (39.5%)	2,827 (41.6%)	1,305 (35.6%)	
**Hypertension**, ***n*** **(%)**				
No	5,422 (52.0%)	3,560 (52.5%)	1,862 (51.1%)	0.166^b^
Yes	4,995 (48.0%)	3,215 (47.5%)	1,780 (48.9%)	
**Diabetes**, ***n*** **(%)**				
No	8,767 (84.6%)	5,746 (85.1%)	3,021 (83.7%)	0.069^b^
Yes	1,597 (15.4%)	1,009 (14.9%)	588 (16.3%)	
**Dyslipidemia**, ***n*** **(%)**				
No	9,146 (89.5%)	6,032 (90.2%)	3,114 (88.1%)	< 0.001^b^
Yes	1,074 (10.5%)	652 (9.8%)	422 (11.9%)	
**Stroke**, ***n*** **(%)**				
No	10,142 (97.4%)	6,632 (97.9%)	3,510 (96.5%)	< 0.001^b^
Yes	268 (2.6%)	140 (2.1%)	128 (3.5%)	
**Cancer**, ***n*** **(%)**				
No	10,285 (99.0%)	6,702 (99.1%)	3,583 (98.8%)	0.102^b^
Yes	106 (1.0%)	61 (0.9%)	45 (1.2%)	
**Lung diseases**, ***n*** **(%)**				
No	9,332 (89.8%)	6,201 (91.7%)	3,131 (86.3%)	< 0.001^b^
Yes	1,061 (10.2%)	564 (8.3%)	497 (13.7%)	
**Heart diseases**, ***n*** **(%)**				
No	9,114 (87.8%)	6,098 (90.2%)	3,016 (83.2%)	< 0.001^b^
Yes	1,269 (12.2%)	661 (9.8%)	608 (16.8%)	
No	9,997 (96.5%)	6,567 (97.3%)	3,430 (94.9%)	< 0.001^b^
Yes	367 (3.5%)	184 (2.7%)	183 (5.1%)	
**Kidney diseases**, ***n*** **(%)**				
No	9,737 (93.8%)	6,434 (95.2%)	3,303 (91.1%)	< 0.001^b^
Yes	643 (6.2%)	321 (4.8%)	322 (8.9%)	
**Stomach diseases**, ***n*** **(%)**				
No	7,972 (76.6%)	5,544 (81.9%)	2,428 (66.7%)	< 0.001^b^
Yes	2,437 (23.4%)	1,227 (18.1%)	1,210 (33.3%)	
**Life satisfaction**, ***n*** **(%)**				
Not at all satisfied	208 (2.2%)	92 (1.5%)	116 (3.5%)	< 0.001^b^
Not very satisfied	1,212 (12.7%)	704 (11.3%)	508 (15.4%)	
Somewhat satisfied	5,685 (59.7%)	3,699 (59.4%)	1,986 (60.3%)	
Very satisfied	2,159 (22.7%)	1,541 (24.7%)	618 (18.8%)	
Completely satisfied	257 (2.7%)	192 (3.1%)	65 (2.0%)	
**Self-rated health**, ***n*** **(%)**				
Poor	672 (6.4%)	565 (8.3%)	107 (2.9%)	< 0.001^b^
Fair	1,626 (15.6%)	1,254 (18.5%)	372 (10.2%)	
Good	5,291 (50.7%)	3,490 (51.5%)	1,801 (49.2%)	
Very good	2,382 (22.8%)	1,248 (18.4%)	1,134 (31.0%)	
Excellent	461 (4.4%)	218 (3.2%)	243 (6.6%)	
**Falls in the past 2 years**, ***n*** **(%)**				
No	8,643 (83.2%)	5,777 (85.7%)	2,866 (78.7%)	< 0.001^b^
Yes	1,741 (16.8%)	967 (14.3%)	774 (21.3%)	
Sleep duration, Median (Q1, Q3)	6.00 (5.00, 8.00)	7.00 (5.00, 8.00)	6.00 (5.00, 7.50)	< 0.001^a^
HDL cholesterol (mg/dl), Median (Q1, Q3)	49 (41, 60)	49 (41, 60)	50 (41, 60)	0.053^a^
LDL cholesterol (mg/dl), Median (Q1, Q3)	113 (92, 135)	113 (92, 135)	113 (92, 136)	0.159^a^
C-reactive protein (mg/L), Median (Q1, Q3)	1.07 (0.57, 2.21)	1.07 (0.56, 2.19)	1.08 (0.59, 2.30)	0.074^a^
Uric acid (mg/dl), Median (Q1, Q3)	4.36 (3.61, 5.24)	4.38 (3.63, 5.29)	4.33 (3.60, 5.16)	0.021^a^

Continuous data are shown as mean median (quartile). Categorical data are shown as n (%).

HDL Cholesterol, high-density lipoprotein cholesterol (mg/dl); LDL cholesterol, low-density lipoprotein cholesterol (mg/dl).

^a^Wilcoxon rank sum test.

^b^Pearson's Chi-squared test.

The longitudinal analysis was based on CHARLS 2011–2012 baseline data with a 10-year follow-up period (median follow-up: 108 months). This cohort included 5,718 individuals, of whom 1,827 (32.0%) developed OA during the follow-up period (Supplementary Figure S1). Baseline analysis showed no significant age difference between those who did and did not develop OA; however, the group that developed OA had higher proportions of women, rural residents, and individuals with lower educational attainment. This group also had significantly higher baseline rates of pulmonary, liver, kidney, and gastrointestinal diseases. Trends in lifestyle factors, biomarker levels, and health-related events were consistent with the baseline findings of the cross-sectional study (Supplementary Table S2).

### Multivariable logistic regression analysis of obesity indexes and IR surrogates in relation to OA

The ORs and 95% CIs for the associations between obesity indices and IR surrogates with OA across three regression models are presented in Table 3. Model 1, a univariate logistic regression, indicated that BFP, LAP, BMI, WHtR, TyG, TyG-WC, TyG-BMI, and TyG-WHtR all showed a significant positive association with OA (all *p* < 0.05). After adjusting for demographic factors, Model 2 demonstrated that BFP, LAP, WHtR, TyG, TyG-WC, TyG-BMI, and TyG-WHtR remained significantly associated with OA (all *p* < 0.05). In Model 3, after full adjustment, BFP (OR = 1.03, 95% CI: 1.02–1.04, *p* < 0.001), LAP (OR = 1.09, 95% CI: 1.04–1.15, *p* < 0.01), BMI (OR = 1.04, 95% CI: 1.02–1.05, *p* < 0.001), WHtR (OR = 1.15, 95% CI: 1.10–1.21, *p* < 0.001), TyG (OR = 1.11, 95% CI: 1.02–1.20, *p* < 0.05), TyG-WC (OR = 1.12, 95% CI: 1.06–1.18, *p* < 0.001), TyG-BMI (OR = 1.16, 95% CI: 1.10–1.22, *p* < 0.001), and TyG-WHtR (OR = 1.23, 95% CI: 1.14–1.32, *p* < 0.001) remained significantly positively associated with OA risk.

**Table 3:** Association of obesity indices and insulin resistance surrogates with OA: a multivariable logistic regression analysis.

**Characteristic**	**OR (95%CI)**, ***p*****-value**		
	**Model 1**	**Model 2**	**Model 3**
BFP (continuous)	1.03 (1.02,1.03) < 0.001	1.03 (1.02,1.03) < 0.001	1.03 (1.02,1.04) < 0.001
**BFP**			
Q1	Reference	Reference	Reference
Q2	1.05 (0.94, 1.18) 0.402	1.04 (0.93, 1.17) 0.498	1.07 (0.94, 1.21) 0.333
Q3	1.32 (1.18, 1.49) < 0.001	1.28 (1.14, 1.44) < 0.001	1.27 (1.10, 1.48) 0.001
Q4	1.68 (1.50, 1.88) < 0.001	1.59 (1.41, 1.78) < 0.001	1.63 (1.39, 1.91) < 0.001
*p* for trend	< 0.001	< 0.001	< 0.001
LAP (standardized)	1.05 (1.01, 1.09) 0.014	1.07 (1.03, 1.11) 0.001	1.09 (1.04,1.15) < 0.001
**LAP**			
Q1	Reference	Reference	Reference
Q2	1.19 (1.06, 1.34) 0.003	1.23 (1.10, 1.38) < 0.001	1.25 (1.11, 1.42) < 0.001
Q3	1.16 (1.03, 1.30) 0.014	1.21 (1.08, 1.36) 0.001	1.27 (1.11, 1.45) < 0.001
Q4	1.30 (1.16, 1.46) < 0.001	1.38 (1.23, 1.55) < 0.001	1.52 (1.31, 1.76) < 0.001
*p* for trend	< 0.001	< 0.001	< 0.001
BMI (continuous)	1.01 (1.00, 1.03) 0.023	1.02 (1.01, 1.03) < 0.001	1.04 (1.02, 1.05) < 0.001
**BMI**			
Q1	Reference	Reference	Reference
Q2	0.96 (0.86, 1.08) 0.485	1.01 (0.90, 1.14) 0.851	1.14 (1.01, 1.28) 0.040
Q3	0.92 (0.82, 1.03) 0.162	0.99 (0.88, 1.11) 0.842	1.12 (0.98, 1.27) 0.085
Q4	1.13 (1.01, 1.27) 0.032	1.22 (1.08, 1.37) 0.001	1.41 (1.23, 1.62) < 0.001
*p* for trend	0.067	0.003	< 0.001
WHtR (standardized)	1.16 (1.11, 1.21) < 0.001	1.10 (1.05, 1.15) < 0.001	1.15 (1.10, 1.21) < 0.001
**WHtR**			
Q1	Reference	Reference	Reference
Q2	1.18 (1.05, 1.33) 0.005	1.16 (1.03, 1.30) 0.014	1.23 (1.09, 1.40) < 0.001
Q3	1.28 (1.14, 1.44) < 0.001	1.23 (1.10, 1.39) < 0.001	1.35 (1.18, 1.53) < 0.001
Q4	1.48 (1.32, 1.66) < 0.001	1.30 (1.15, 1.47) < 0.001	1.48 (1.29, 1.70) < 0.001
*p* for trend	< 0.001	< 0.001	< 0.001
TyG (continuous)	1.07 (1.01, 1.14) 0.025	1.07 (1.00, 1.13) 0.037	1.11 (1.02, 1.20) 0.012
**TyG**			
Q1	Reference	Reference	Reference
Q2	1.04 (0.93, 1.17) 0.494	1.02 (0.91, 1.15) 0.709	1.01 (0.89, 1.14) 0.849
Q3	1.19 (1.06, 1.33) 0.003	1.15 (1.02, 1.29) 0.021	1.19 (1.05, 1.35) 0.008
Q4	1.15 (1.02, 1.28) 0.020	1.13 (1.01, 1.27) 0.038	1.18 (1.02, 1.36) 0.024
*p* for trend	0.003	0.010	0.004
TyG-WC (standardized)	1.04 (1.00, 1.08) 0.045	1.05 (1.01, 1.10) 0.011	1.12 (1.06, 1.18) < 0.001
**TyG-WC**			
Q1	Reference	Reference	Reference
Q2	1.09 (0.98, 1.23) 0.123	1.10 (0.98, 1.24) 0.094	1.17 (1.04, 1.33) 0.010
Q3	1.10 (0.98, 1.24) 0.091	1.12 (0.99, 1.25) 0.062	1.24 (1.09, 1.41) 0.001
Q4	1.14 (1.01, 1.27) 0.028	1.17 (1.04, 1.31) 0.010	1.35 (1.17, 1.56) < 0.001
*p* for trend	0.034	0.013	< 0.001
TyG-BMI (standardized)	1.06 (1.01, 1.10) 0.008	1.08 (1.03, 1.12) < 0.001	1.16 (1.10, 1.22) < 0.001
**TyG-BMI**			
Q1	Reference	Reference	Reference
Q2	1.06 (0.95, 1.19) 0.281	1.10 (0.98, 1.24) 0.104	1.23 (1.08, 1.39) 0.001
Q3	1.07 (0.96, 1.20) 0.233	1.11 (0.99, 1.25) 0.072	1.28 (1.12, 1.46) < 0.001
Q4	1.14 (1.02, 1.27) 0.026	1.20 (1.07, 1.35) 0.003	1.43 (1.24, 1.66) < 0.001
*p* for trend	0.032	0.004	< 0.001
TyG-WHtR (continuous)	1.18 (1.12, 1.24) < 0.001	1.12 (1.06, 1.19) < 0.001	1.23 (1.14, 1.32) < 0.001
**TyG_WHtR**			
Q1	Reference	Reference	Reference
Q2	1.24 (1.10, 1.39) < 0.001	1.21 (1.07, 1.36) 0.002	1.30 (1.15, 1.47) < 0.001
Q3	1.28 (1.14, 1.43) < 0.001	1.21 (1.08, 1.37) 0.001	1.35 (1.18, 1.54) < 0.001
Q4	1.45 (1.29, 1.62) < 0.001	1.32 (1.17, 1.49) < 0.001	1.56 (1.35, 1.81) < 0.001
*p* for trend	< 0.001	< 0.001	< 0.001

OR, odds ratio; CI, confidence interval; BFP, body fat percentage; LAP, lipid accumulation product; BMI, body mass index; WHtR, waist-to-height ratio; TyG, triglyceride-glucose; TyG-WC, TyG with waist circumference.

Model 1: unadjusted for covariates.

Model 2: adjusted for Age, Sex, Education, Marital status, and Residence place.

Model 3: additionally adjusted for Drink, Smoke, Hypertension, Diabetes, Dyslipidemia, Stroke, Cancer, Lung diseases, Heart diseases, Liver diseases, Kidney diseases, Stomach diseases, Sleep duration, Life satisfaction, Self-rated health, Falls in the past 2 years, HDL Cholesterol (mg/dl), LDL Cholesterol (mg/dl), C-Reactive Protein (mg/L), and Uric acid (mg/dl), beyond the variables in Model 2.

Since BFP and LAP calculations already account for gender, it was not adjusted for in Models 2 and 3.

When these indices were divided into quartiles, the highest quartile showed a significantly higher prevalence of OA compared to the lowest quartile. The ORs for the fourth quartile were as follows: BFP, 1.63 (95% CI: 1.39–1.91); LAP, 1.52 (95% CI: 1.31–1.76); BMI, 1.41 (95% CI: 1.23–1.62); WHtR, 1.48 (95% CI: 1.29–1.70); TyG, 1.18 (95% CI: 1.02–1.36); TyG-WC, 1.35 (95% CI: 1.17–1.56); TyG-BMI, 1.43 (95% CI: 1.24–1.66); and TyG-WHtR, 1.56 (95% CI: 1.35–1.81). All indices exhibited a significant trend (*p* for trend < 0.05).

### Non-linear associations between obesity indexes and IR surrogates with OA

**Figure 3:**
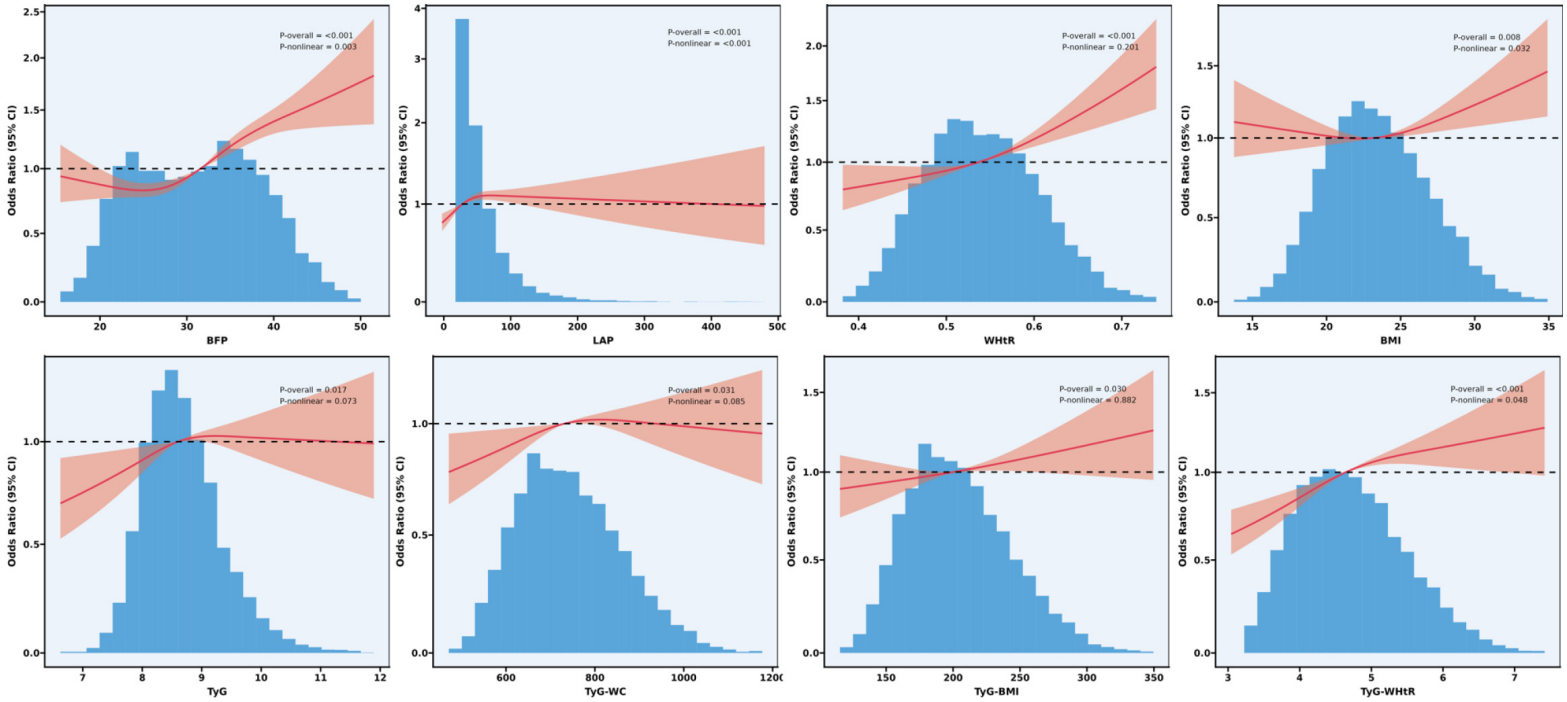
Non-linear association of obesity indices and surrogate markers of IR surrogates with OA: restricted cubic spline.

Furthermore, the RCS analysis (Figure 3) revealed a significant positive association between various obesity indices and OA (*p* overall < 0.05). Specifically, the BFP, LAP, BMI and TyG-WHtR showed a significant non-linear relationship with OA (*p* non-linear < 0.05), whereas TyG, WHtR, TyG-WC, and TyG-BMI exhibited predominantly non-significant non-linear associations (*p* non-linear >0.05), suggesting linear relationships.

### Multivariate logistic regression analysis of obesity indices and IR surrogates in different gender subgroups and their association with OA

To further evaluate the relationship between obesity indices, the TyG index, and its derived indices with OA, we conducted a sex-stratified analysis and performed interaction tests (Table 4). The results revealed a significant interaction between BFP and sex (*p* = 0.038), with a more pronounced effect observed in females. In contrast, no significant interaction effects with sex were observed for LAP, BMI, WHtR, TyG, TyG-WC, TyG-BMI, or TyG-WHtR (all interaction *p*-values >0.05).

**Table 4:** Sex-specific associations of obesity indices and insulin resistance surrogates with OA: multivariable logistic regression analysis.

**Characteristic**	**Subgroup**	**OR (95% CI)^a^**	***p*-value**	***p* for interaction**
BFP	**Sex**			0.038
	Male	1.02 (1.00–1.04)	0.02	
	Female	1.04 (1.02–1.05)	< 0.001	
LAP	**Sex**			0.103
	Male	1.15 (1.07–1.24)	< 0.001	
	Female	1.01 (0.95–1.08)	0.71	
BMI	**Sex**			0.282
	Male	1.03 (1.00–1.05)	0.02	
	Female	1.04 (1.02–1.06)	< 0.001	
WHtR	**Sex**			0.909
	Male	1.19 (1.09–1.30)	< 0.001	
	Female	1.15 (1.07–1.24)	< 0.001	
TyG	**Sex**			0.083
	Male	1.23 (1.10–1.39)	< 0.001	
	Female	1.00 (0.89–1.12)	0.99	
TyG-WC	**Sex**			0.525
	Male	1.12 (1.03–1.22)	0.007	
	Female	1.12 (1.04–1.20)	0.003	
TyG_BMI	**Sex**			0.247
	Male	1.16 (1.07–1.27)	0.001	
	Female	1.15 (1.07–1.23)	< 0.001	
TyG_WHtR	**Sex**			0.909
	Male	1.26 (1.13–1.42)	< 0.001	
	Female	1.21 (1.10–1.32)	< 0.001	

OR, odds ratio; CI, confidence interval; BFP, body fat percentage; LAP, lipid accumulation product; BMI, body mass index; WHtR, waist-to-height ratio; TyG, triglyceride-glucose; TyG-WC, TyG with waist circumference.

^a^Adjusted for Age, Education, Marital status, Residence place, Drink, Smoke, Hypertension, Diabetes, Dyslipidemia, Stroke, Cancer, Lung diseases, Heart diseases, Liver diseases, Kidney diseases, Stomach diseases, Sleep duration, Life satisfaction, Self-rated health, Falls in the past 2 years, HDL Cholesterol (mg/dl), LDL Cholesterol (mg/dl), C-Reactive Protein (mg/L), and Uric acid (mg/dl).

### Comparison of obesity indices, TyG index, and its derivatives in predicting OA

**Figure 4:**
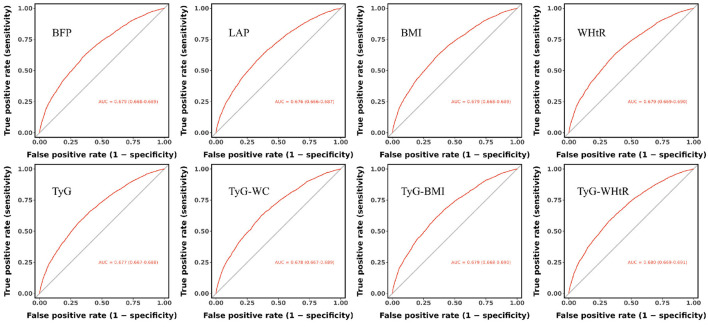
Comparative predictive performance of obesity indices and IR surrogates for OA: receiver operating characteristic curve analysis.

The results of the ROC analysis are presented in Figure 4. The area under the curve (AUC) values for BFP, LAP, BMI, WHtR, TyG, TyG-WC, TyG-BMI, and TyG-WHtR were 0.679, 0.676, 0.679, 0.679, 0.677, 0.678, 0.679, and 0.680, respectively. Among these, TyG-WHtR exhibited the highest AUC, suggesting superior diagnostic performance in predicting OA (Figure 4).

Additionally, sensitivity and specificity analyses (Table 5) further supported the strong predictive value of TyG-WHtR for OA risk.

**Table 5:** Sensitivity, specificity, and optimal thresholds of obesity index and insulin resistance surrogates in predicting OA.

**Characteristic**	**Sensitivity**	**Specificity**	**Cut-off**
BFP	0.661	0.599	0.332
LAP	0.578	0.680	0.363
BMI	0.650	0.613	0.337
WHtR	0.600	0.661	0.356
TyG	0.591	0.667	0.360
TyG-WC	0.627	0.634	0.346
TyG-BMI	0.645	0.614	0.339
TyG-WHtR	0.681	0.579	0.365

BFP, body fat percentage; LAP, lipid accumulation product; BMI, body mass index; WHtR, waist-to-height ratio; TyG, triglyceride-glucose; TyG-WC, TyG with waist circumference.

Adjusted for Age, sex, Education, Marital status, Residence place, Drink, Smoke, Hypertension, Diabetes, Dyslipidemia, Stroke, Cancer, Lung diseases, Heart diseases, Liver diseases, Kidney diseases, Stomach diseases, Sleep duration, Life satisfaction, Self-rated health, Falls in the past 2 years, HDL Cholesterol (mg/dl), LDL Cholesterol (mg/dl), C-Reactive Protein (mg/L), and Uric acid (mg/dl)

### Multivariable cox regression analysis of TyG-WHtR in relation to OA

The results of the multivariable Cox regression analysis revealed a significant positive association between the TyG-WHtR, analyzed as a continuous variable, and the risk of OA. In the unadjusted Model 1, a one-unit increase in TyG-WHtR was associated with a 10% elevated risk of OA (HR = 1.10, 95% CI: 1.03–1.16, *p* = 0.003). Following full adjustment for covariates in Model 3, this risk increased to 20% (HR = 1.20, 95% CI: 1.11–1.31, *p* < 0.001). When TyG-WHtR was analyzed in quartiles using Q1 as the reference group, the risk of OA was significantly higher in Q2 (HR = 1.19, 95% CI: 1.04–1.37, *p* = 0.011), Q3 (HR = 1.22, 95% CI: 1.05–1.41, *p* = 0.008), and Q4 (HR = 1.46, 95% CI: 1.24–1.72, *p* < 0.001), representing increases of 19%, 22%, and 46%, respectively. Furthermore, trend tests indicated a significant dose-response relationship (*p* < 0.05; Table 6).

**Table 6:** Association of TyG-WHtR and OA: a multivariable cox regression analysis.

**Characteristic**	**HR (95%CI)**, ***p*****-value**		
	**Model 1**	**Model 2**	**Model 3**
TyG_WHtR (continuous)	1.10 (1.03, 1.16) 0.003	1.08 (1.01, 1.15) 0.018	1.20 (1.11, 1.31) < 0.001
**TyG_WHtR**			
Q1	Reference	Reference	Reference
Q2	1.11 (0.97, 1.27) 0.121	1.11 (0.97, 1.27) 0.128	1.19 (1.04, 1.37) 0.011
Q3	1.08 (0.94, 1.23) 0.260	1.08 (0.95, 1.24) 0.252	1.22 (1.05, 1.41) 0.008
Q4	1.24 (1.08, 1.41) 0.001	1.20 (1.05, 1.38) 0.008	1.46 (1.24, 1.72) < 0.001
*p* for trend	0.004	0.017	< 0.001

HR, hazard ratio; CI, confidence interval; TyG-WHtR, TyG with waist-to-height ratio.

Model 1: unadjusted for covariates.

Model 2: adjusted for Age, Sex, Education, Marital status, and Residence place.

Model 3: additionally adjusted for Drink, Smoke, Hypertension, Diabetes, Dyslipidemia, Stroke, Cancer, Lung diseases, Heart diseases, Liver diseases, Kidney diseases, Stomach diseases, Sleep duration, Life satisfaction, Self-rated health, Falls in the past 2 years, HDL Cholesterol (mg/dl), LDL Cholesterol (mg/dl), C-Reactive Protein (mg/L), and Uric acid (mg/dl), beyond the variables in Model 2.

### Multivariable cox regression analysis of TyG-WHtR and OA in different subgroups and their association

**Figure 5:**
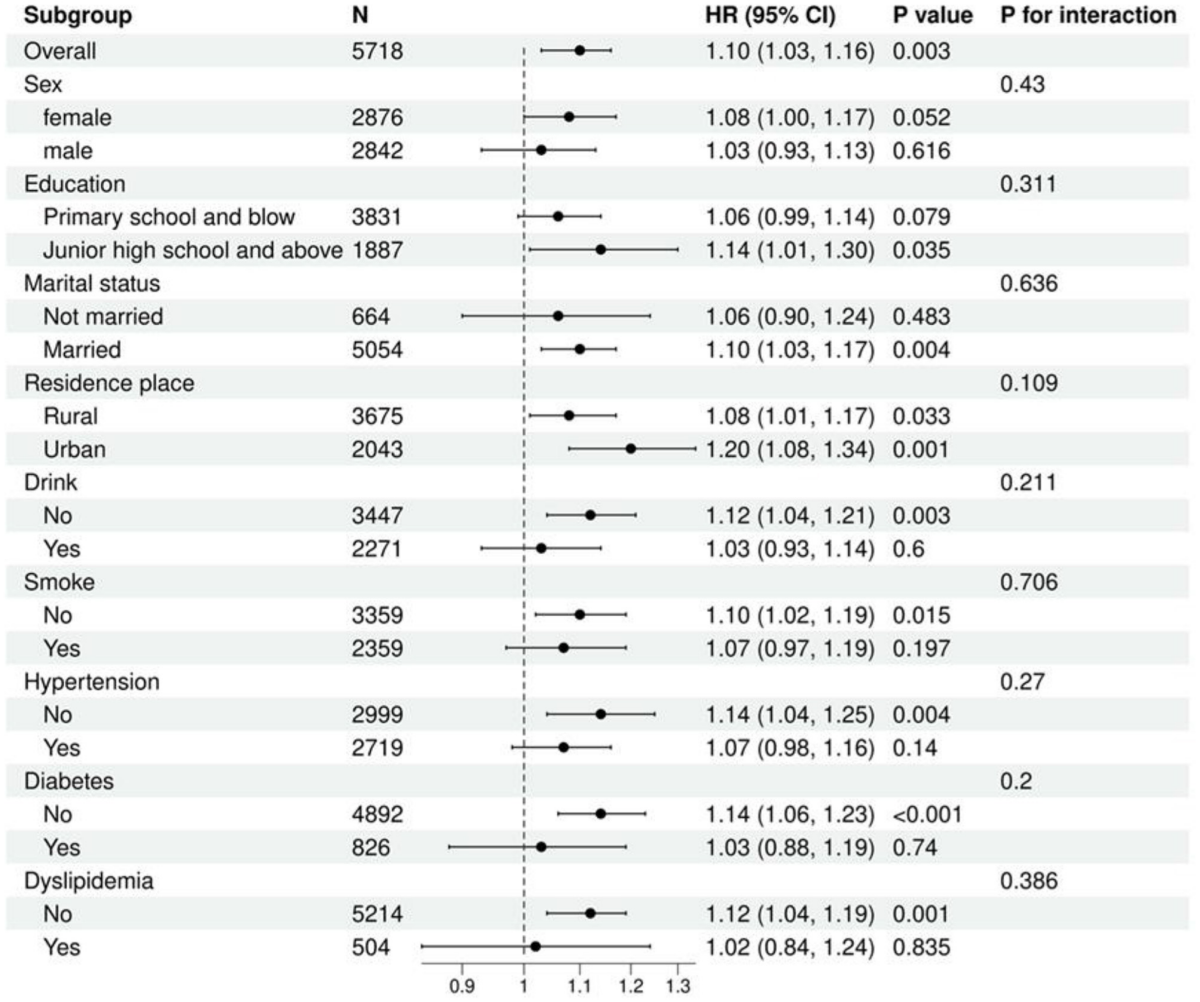
Multivariable cox regression analysis of TyG-WHtR and OA in different subgroups.

We used Cox regression models to examine the relationship between TyG-WHtR and the risk of OA. Subgroup analyses were also conducted, revealing no statistically significant interactions in the subgroups of sex, education, marital status, residence place, drink, smoke, hypertension, diabetes, and dyslipidemia (Figure 5).

## Discussion

In light of China's aging population and the rising prevalence of OA, this study aims to explore the relationship between traditional obesity indicators (including BFP, LAP, BMI, and WHtR) and IR surrogate (TyG and its derivatives: TyG-WC, TyG-BMI, and TyG-WHtR) with OA risk, as well as to evaluate the clinical utility of these indicators in OA diagnosis. Our findings indicate that TyG-WHtR exhibits significant diagnostic performance and robust reliability in OA risk assessment, positioning it as a potentially effective tool for clinical screening and early diagnosis.

This study analyzed cross-sectional data from 10,457 participants, including 3,667 individuals diagnosed with OA. In a fully adjusted multivariable model, all indicators (analyzed as continuous variables) were positively associated with OA risk (all *p* < 0.05). When categorized into quartiles, the highest quartile (Q4) showed a significantly increased risk of OA compared to the lowest quartile (Q1) in the fully adjusted model (all *p* < 0.05). ROC curve analysis of occupational characteristics identified TyG-WHtR as the strongest predictor of OA risk, with an AUC of 0.680. The sensitivity and specificity were 0.681 and 0.579, respectively. Despite its moderate specificity, the relatively high sensitivity suggests that TyG-WHtR is a valuable screening tool for identifying high-risk populations who may benefit from early intervention.

Further RCS analysis revealed significant positive correlations between several metabolic indices and OA. Specifically, BFP, LAP, BMI, and TyG-WHtR demonstrated significant nonlinear relationships with OA. In contrast, TyG, WHtR, TyG-WC, and TyG-BMI exhibited predominantly nonsignificant nonlinear associations, suggesting a potential linear relationship with OA. In sex-specific subgroup analyses, a significant interaction was observed between BFP and gender. This is consistent with previous reports of a higher prevalence of symptomatic knee OA among women in China (39). Studies suggest that obesity may exacerbate knee pain in middle-aged women through hormonal mechanisms, thereby increasing OA risk (40). No significant interactions were found between gender and the other indicators (LAP, BMI, WHtR, TyG, TyG-WC, TyG-BMI, and TyG-WHtR).

To validate the robustness of TyG-WHtR as a predictive marker, we conducted a subsequent longitudinal cohort study. Multivariable Cox regression analysis was performed on follow-up data from 5,718 participants (including 1,827 OA individuals) over a median follow-up period of 108 months. The results showed that TyG-WHtR, as a continuous variable, was significantly positively associated with OA risk; each unit increase in TyG-WHtR was associated with a 20% increase in OA risk. When analyzed by quartiles, the Q4 group had a 46% higher risk of OA compared to the Q1 group, with a significant dose-response relationship across quartiles (all *p* < 0.05). Subgroup analyses revealed no significant effect modification, further confirming the broad applicability and robustness of TyG-WHtR as a predictor of OA risk across different populations.

OA is a leading cause of disability among middle-aged and elderly populations, imposing substantial physical suffering on patients and escalating healthcare costs due to prolonged disability care (41). The relationship between obesity and OA extends beyond the traditional mechanical load theory (42). Recent studies have increasingly highlighted the critical role of metabolic abnormalities in the onset and progression of OA, particularly the synergistic effects of insulin resistance and systemic inflammation (43, 44). Metabolic syndrome, a major comorbidity of obesity, accelerates joint degeneration through various molecular mechanisms, leading to a distinct metabolic OA phenotype (45, 46). This metabolic form of OA affects not only cartilage but also involves multiple joint tissues, including the synovium, subchondral bone, tendons, ligaments, menisci, and adipose pads (47).

As the primary load-bearing tissue in joints, cartilage is directly impacted by metabolic disturbances (48). Insulin resistance and hyperinsulinemia alter chondrocyte metabolism, promoting catabolic activity (49). In hyperglycemic environments, the accumulation of advanced glycation end-products exacerbates this process. These products bind to the RAGE receptor on chondrocyte surfaces, triggering inflammatory pathways and increasing the expression of matrix metalloproteinases, which accelerate the degradation of the cartilage matrix (50). Additionally, in metabolic OA, the subchondral bone undergoes significant remodeling, with fibrotic metabolic dysfunction-associated steatotic liver disease closely linked to subchondral bone loss through the influence of systemic inflammatory mediators and metabolic byproducts that regulate bone remodeling (51).

The synovium, which functions as a cushioning tissue in the joint, plays a critical role in the pathology of OA (52). Obesity and metabolic disorders significantly impact the inflammatory state and function of the synovium. Synovial inflammation occurs at all stages of OA and is closely associated with disease progression (53). Resistin, an important adipokine, upregulates fatty acid oxidation (FAO) in synovial cells via the CAP1/PKA/CREB signaling pathway, thereby promoting inflammation and a catabolic phenotype that further exacerbates the progression of metabolic syndrome-related knee OA (MetS-KOA) (54). Concurrently, declines in bone density and alterations in bone microstructure are particularly prominent in metabolic OA, and these changes not only impact the joint's biomechanical environment but may also accelerate cartilage degeneration (55). Joint-associated structures, including tendons, ligaments, and menisci, which are fibrocartilaginous tissues, may undergo similar changes due to metabolic disturbances, leading to alterations in extracellular matrix composition, a reduction in mechanical properties, and a diminished capacity for repair (25, 51).

Adipose pads, specialized fat tissues within the joint, serve as mechanical buffers but may also influence the joint environment by secreting adipokines and inflammatory mediators locally, thereby affecting the onset and progression of OA (56, 57). Furthermore, obesity-induced sarcopenia has become a significant risk factor for various adverse health outcomes (58). The deterioration of muscle tissue integrity, characterized by persistent muscle loss, intramuscular lipid accumulation, and connective tissue deposition, is a hallmark of metabolic dysfunction, and these changes not only affect joint stability and load distribution but may also exacerbate OA progression through the secretion of myokines that modulate local inflammatory responses (59).

Traditional obesity indices, including BFP, LAP, BMI, and WHtR, are widely used in metabolic and musculoskeletal research (60). Elevated BMI strongly correlates with knee OA (61), whereas BFP better reflects adiposity-related risks, associating with heightened pain sensitivity and reduced tibial cartilage thickness (62, 63). LAP, which integrates waist circumference and triglycerides, effectively captures visceral adiposity and lipid dysmetabolism, outperforming BMI in metabolic syndrome diagnosis (64, 65). Additionally, a study based on the National Health and Nutrition Examination Survey (NHANES) found a distinct threshold effect of LAP on OA risk (66). The triglyceride-glucose (TyG) index, a cost-effective IR surrogate, and its derivatives (TyG-WC, TyG-BMI, TyG-WHtR) robustly predict prediabetes, cardiovascular risk, and OA incidence (67–69).

Strengths of this study include a comprehensive evaluation of obesity and IR surrogates, consistent validation across subgroups, and mechanistic integration of obesity-IR interactions in OA pathogenesis. However, several limitations must be acknowledged. First, while self-reported OA diagnosis demonstrated 85% agreement with clinical assessment (70), this method is susceptible to recall and social desirability biases, which may compromise diagnostic accuracy and, consequently, the reliability of our findings. Second, the CHARLS database lacks detailed information on OA lesion locations and severity, restricting more nuanced analyses of patient subgroups. Future studies should incorporate clinical symptoms, physical examinations, and imaging data to improve diagnostic precision and validate the robustness of these associations. Third, with the exception of the TyG-WHtR indicator, which was also analyzed in a longitudinal cohort, all other indicators were assessed solely using cross-sectional data. This design limits the ability to establish causal inferences for the majority of the obesity and IR surrogate markers in relation to OA risk. Future longitudinal studies are warranted to elucidate these potential causal relationships. Finally, certain unmeasured confounding factors, such as occupational history, medication use, and genetic predisposition, which are not available in the CHARLS dataset, could influence the results. Incorporating these variables in future analyses would improve the precision and reliability of the findings.

## Conclusion

Obesity indices and IR surrogates were significantly associated with the risk of OA, with the TyG-WHtR demonstrating the strongest predictive value. Our findings indicate that TyG-WHtR could serve as a promising screening tool for OA. Furthermore, these results underscore obesity and IR as modifiable risk factors, providing a theoretical foundation for early prevention. Timely lifestyle interventions and metabolic regulation may help delay the onset and slow the progression of OA. To enhance the generalizability and clinical applicability of these findings, further validation in large-scale, multi-ethnic cohorts, complemented by mechanistic investigations, is warranted. Such efforts could ultimately facilitate the development of personalized strategies for OA prevention and management.

## Data Availability

The datasets presented in this study can be found in online repositories. The names of the repository/repositories and accession number(s) can be found below: https://charls.pku.edu.cn/.

## Supplementary material

SUPPLEMENTARY FIGURE S1 Population screening process based on multivariable Cox regression analysis.

SUPPLEMENTARY TABLE S1 The variance inflation factor and tolerance values of each indicator.

SUPPLEMENTARY TABLE S2 Baseline characteristics of multivariate cox regression analysis.
