# Supplementary figures and images for: Association between obesity indices, insulin resistance markers, and osteoarthritis in middle-aged and elderly Chinese adults
Source: Front Nutr. 2025 Oct 31;12:1627421. doi: 10.3389/fnut.2025.1627421 (PMC12617301; doi:10.3389/fnut.2025.1627421)

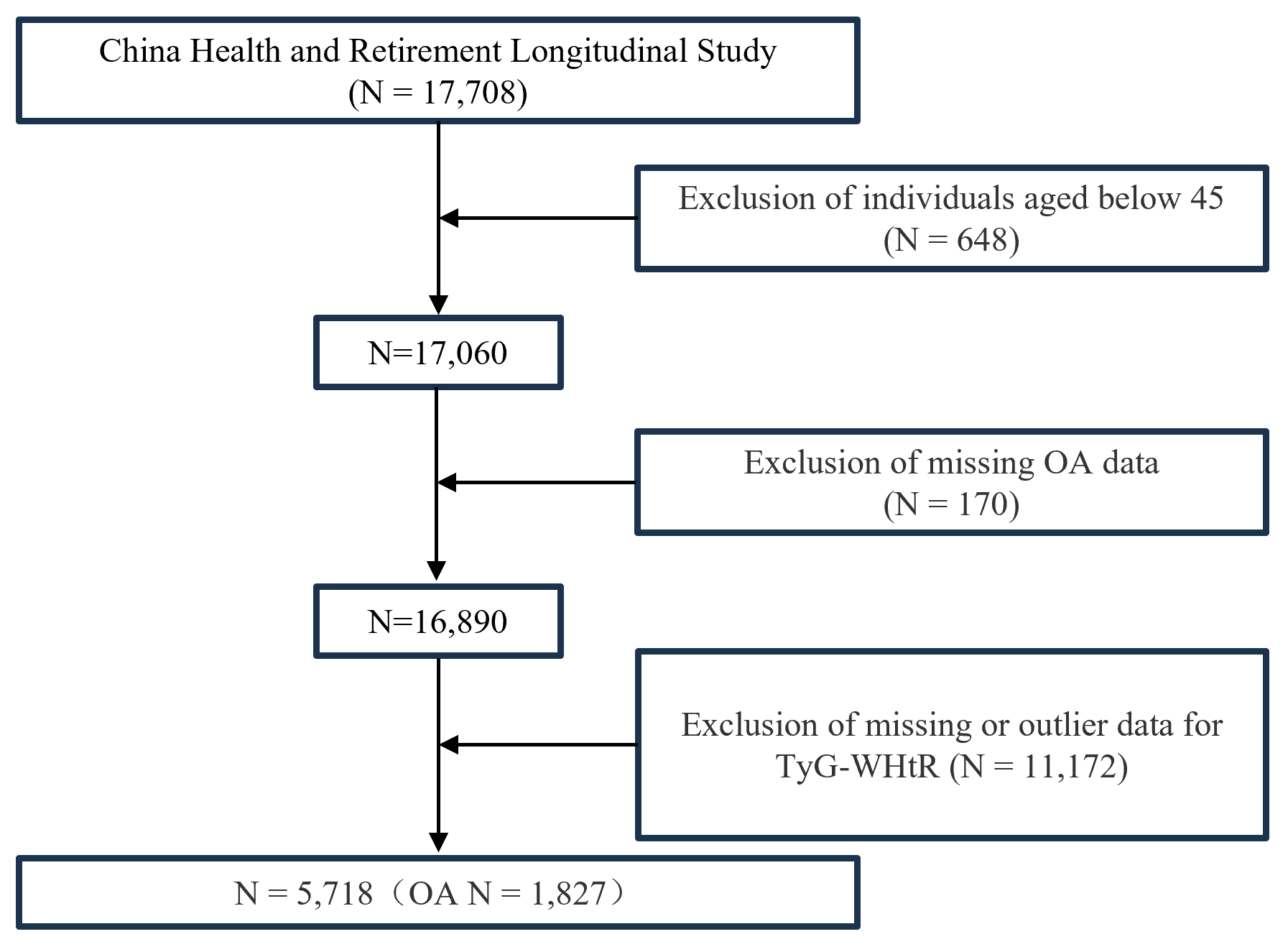

Supplement: SUPPLEMENTARY FIGURE S1 — Population screening process based on multivariable Cox regression analysis. [file Image_1.jpg]
