# Supplementary material for: Association between obesity indices, insulin resistance markers, and osteoarthritis in middle-aged and elderly Chinese adults
Source: Front Nutr. 2025 Oct 31;12:1627421. doi: 10.3389/fnut.2025.1627421 (PMC12617301; doi:10.3389/fnut.2025.1627421)
Supplement: SUPPLEMENTARY TABLE S1 — The variance inflation factor and tolerance values of each indicator. [file Table_1.pdf]

LAP was analyzed as a standardized continuous variable

Model 2 : Variance Inflation Factor and Tolerance

Term	VIF	VIF_CI_low	VIF_CI_high	SE_factor	Tolerance	Tolerance_CI_low	Tolerance_CI_high
LAP_normalized	1.015416	1.004284	1.055478	1.007679	0.9848176	0.9474383	0.9957343
Age	1.188471	1.163863	1.216775	1.090170	0.8414171	0.8218446	0.8592076
Education	1.132056	1.109936	1.158626	1.063981	0.8833487	0.8630914	0.9009527
Marital.status	1.107153	1.086248	1.133124	1.052213	0.9032177	0.8825156	0.9206000
Residence.place	1.057634	1.039786	1.083488	1.028413	0.9455065	0.9229452	0.9617359

LAP was analyzed as a standardized continuous variable

Model 3 : Variance Inflation Factor and Tolerance

Term	VIF	VIF_CI_low	VIF_CI_high	SE_factor	Tolerance	Tolerance_CI_low	Tolerance_CI_high
LAP_normalized	1.418954	1.385275	1.455578	1.191199	0.7047443	0.6870125	0.7218783
Age	1.338750	1.308174	1.372361	1.157044	0.7469652	0.7286712	0.7644245
Education	1.177005	1.152940	1.204857	1.084899	0.8496137	0.8299739	0.8673475
Marital.status	1.119276	1.097814	1.145446	1.057958	0.8934351	0.8730225	0.9109011
Residence.place	1.095190	1.074962	1.120876	1.046513	0.9130835	0.8921589	0.9302653
Drink	1.318877	1.289076	1.351750	1.148424	0.7582208	0.7397818	0.7757493
Smoke	1.354486	1.323297	1.388684	1.163824	0.7382873	0.7201063	0.7556880
Hypertension	1.149833	1.126953	1.176837	1.072303	0.8696913	0.8497355	0.8873483
Diabetes	1.102926	1.082286	1.128743	1.050203	0.9066792	0.8859411	0.9239702
Dyslipidemia	1.127756	1.105888	1.154140	1.061959	0.8867166	0.8664462	0.9042503
Stroke	1.035328	1.019801	1.063031	1.017511	0.9658774	0.9407063	0.9805835
Cancer	1.019181	1.006820	1.053951	1.009545	0.9811797	0.9488106	0.9932266
Lung.diseases	1.050170	1.032998	1.076279	1.024778	0.9522269	0.9291273	0.9680565
Heart.diseases	1.101624	1.081052	1.127417	1.049583	0.9077508	0.8869831	0.9250248
Liver.diseases	1.021963	1.008859	1.054455	1.010922	0.9785086	0.9483576	0.9912191
Kidney.diseases	1.029886	1.015180	1.058839	1.014833	0.9709813	0.9444307	0.9850470
Stomach.diseases	1.052899	1.035483	1.078864	1.026109	0.9497583	0.9269008	0.9657327
Sleep.duration	1.040713	1.024511	1.067624	1.020153	0.9608800	0.9366590	0.9760757
Life.satisfaction	1.107249	1.086386	1.133151	1.052259	0.9031392	0.8824952	0.9204832
Self.rated.health	1.226787	1.200651	1.256326	1.107604	0.8151378	0.7959716	0.8328815
Falls.in.the.past.two.years	1.033886	1.018561	1.061865	1.016802	0.9672247	0.9417389	0.9817777
HDL.Cholesterol.mg.dL.	1.287914	1.259331	1.319648	1.134863	0.7764495	0.7577781	0.7940727
LDL.Cholesterol.mg.dL.	1.051874	1.034548	1.077889	1.025609	0.9506843	0.9277391	0.9666059
C.Reactive.Protein.mg.L.	1.022202	1.009039	1.054534	1.011040	0.9782799	0.9482861	0.9910418
Uric.acid.mg.dL.	1.185621	1.161190	1.213755	1.088862	0.8434398	0.8238896	0.8611854

LAP was treated as a categorical variable

Model 2 : Variance Inflation Factor and Tolerance

Term	VIF	VIF_CI_low	VIF_CI_high	SE_factor	Tolerance	Tolerance_CI_low	Tolerance_CI_high
LAP_cat	1.026576	1.012444	1.056757	1.013201	0.9741115	0.9462911	0.9877086
Age	1.190118	1.165445	1.218471	1.090925	0.8402527	0.8207007	0.8580414
Education	1.131827	1.109722	1.158385	1.063874	0.8835272	0.8632706	0.9011266
Marital.status	1.107965	1.087022	1.133948	1.052599	0.9025555	0.8818749	0.9199443
Residence.place	1.068315	1.049677	1.093944	1.033593	0.9360539	0.9141235	0.9526738

LAP was treated as a categorical variable

Model 3 : Variance Inflation Factor and Tolerance

Term	VIF	VIF_CI_low	VIF_CI_high	SE_factor	Tolerance	Tolerance_CI_low	Tolerance_CI_high
LAP_cat	1.620916	1.579540	1.665245	1.273152	0.6169353	0.6005123	0.6330956
Age	1.341401	1.310726	1.375103	1.158188	0.7454895	0.7272182	0.7629359
Education	1.176425	1.152389	1.204252	1.084631	0.8500330	0.8303910	0.8677626
Marital.status	1.120341	1.098832	1.146532	1.058462	0.8925853	0.8721956	0.9100575
Residence.place	1.099509	1.079053	1.125260	1.048575	0.9094967	0.8886838	0.9267390
Drink	1.322550	1.292611	1.355552	1.150022	0.7561150	0.7377067	0.7736279
Smoke	1.400143	1.367194	1.436048	1.183276	0.7142129	0.6963555	0.7314252
Hypertension	1.165317	1.141759	1.192789	1.079498	0.8581359	0.8383713	0.8758415
Diabetes	1.079888	1.060543	1.105415	1.039177	0.9260217	0.9046373	0.9429134
Dyslipidemia	1.125200	1.103457	1.151512	1.060754	0.8887308	0.8684233	0.9062425
Stroke	1.035442	1.019901	1.063117	1.017567	0.9657712	0.9406300	0.9804869
Cancer	1.019758	1.007233	1.053971	1.009831	0.9806246	0.9487931	0.9928186
Lung.diseases	1.050185	1.033014	1.076287	1.024785	0.9522131	0.9291203	0.9680411
Heart.diseases	1.103345	1.082687	1.129165	1.050402	0.9063348	0.8856105	0.9236282
Liver.diseases	1.021858	1.008780	1.054411	1.010870	0.9786099	0.9483965	0.9912959
Kidney.diseases	1.029716	1.015040	1.058711	1.014749	0.9711420	0.9445452	0.9851827
Stomach.diseases	1.053256	1.035812	1.079198	1.026283	0.9494366	0.9266139	0.9654263
Sleep.duration	1.041079	1.024838	1.067942	1.020333	0.9605417	0.9363807	0.9757644
Life.satisfaction	1.110463	1.089441	1.136427	1.053785	0.9005249	0.8799510	0.9179019
Self.rated.health	1.226855	1.200722	1.256391	1.107635	0.8150922	0.7959305	0.8328325
Falls.in.the.past.two.years	1.035321	1.019797	1.063018	1.017507	0.9658840	0.9407177	0.9805873
HDL.Cholesterol.mg.dL.	1.383338	1.351038	1.418611	1.176154	0.7228889	0.7049148	0.7401717
LDL.Cholesterol.mg.dL.	1.077868	1.058647	1.103389	1.038204	0.9277576	0.9062990	0.9446023
C.Reactive.Protein.mg.L.	1.022854	1.009537	1.054765	1.011363	0.9776565	0.9480784	0.9905528
Uric.acid.mg.dL.	1.183737	1.159390	1.211803	1.087997	0.8447821	0.8252166	0.8625223

BFP was treated as a continuous variable

Model 2 : Variance Inflation Factor and Tolerance

Term	VIF	VIF_CI_low	VIF_CI_high	SE_factor	Tolerance	Tolerance_CI_low	Tolerance_CI_high
BFP	1.053846	1.036313	1.079846	1.026570	0.9489051	0.9260584	0.9649597
Age	1.190813	1.166106	1.219195	1.091244	0.8397621	0.8202131	0.8575546
Education	1.156288	1.133068	1.183559	1.075308	0.8648367	0.8449091	0.8825596
Marital.status	1.114077	1.092823	1.140198	1.055499	0.8976038	0.8770407	0.9150611
Residence.place	1.068440	1.049791	1.094074	1.033654	0.9359436	0.9140146	0.9525702

BFP was treated as a continuous variable

Model 3 : Variance Inflation Factor and Tolerance

Term	VIF	VIF_CI_low	VIF_CI_high	SE_factor	Tolerance	Tolerance_CI_low	Tolerance_CI_high
BFP	1.969153	1.914616	2.026942	1.403265	0.5078325	0.4933540	0.5222979
Age	1.350601	1.319563	1.384654	1.162154	0.7404110	0.7222022	0.7578265
Education	1.186066	1.161617	1.214215	1.089067	0.8431231	0.8235774	0.8608692
Marital.status	1.122236	1.100631	1.148479	1.059356	0.8910783	0.8707171	0.9085696
Residence.place	1.105174	1.084417	1.131034	1.051273	0.9048349	0.8841465	0.9221541
Drink	1.381802	1.349555	1.417024	1.175501	0.7236927	0.7057044	0.7409850
Smoke	1.788614	1.740886	1.839418	1.337391	0.5590920	0.5436504	0.5744201
Hypertension	1.165301	1.141740	1.192778	1.079491	0.8581476	0.8383789	0.8758564
Diabetes	1.062562	1.044372	1.088210	1.030807	0.9411213	0.9189404	0.9575133
Dyslipidemia	1.129094	1.107164	1.155513	1.062589	0.8856655	0.8654164	0.9032088
Stroke	1.035561	1.020002	1.063222	1.017625	0.9656600	0.9405370	0.9803898
Cancer	1.019213	1.006842	1.053952	1.009561	0.9811487	0.9488102	0.9932041
Lung.diseases	1.051852	1.034528	1.077869	1.025598	0.9507039	0.9277568	0.9666243
Heart.diseases	1.106275	1.085462	1.132157	1.051796	0.9039345	0.8832698	0.9212670
Liver.diseases	1.021688	1.008651	1.054369	1.010786	0.9787726	0.9484347	0.9914229
Kidney.diseases	1.030091	1.015351	1.058985	1.014934	0.9707882	0.9443005	0.9848815
Stomach.diseases	1.053765	1.036274	1.079690	1.026531	0.9489780	0.9261918	0.9649955
Sleep.duration	1.040729	1.024525	1.067639	1.020161	0.9608647	0.9366461	0.9760617
Life.satisfaction	1.109045	1.088091	1.134984	1.053112	0.9016764	0.8810695	0.9190408
Self.rated.health	1.225702	1.199611	1.255204	1.107114	0.8158587	0.7966832	0.8336035
Falls.in.the.past.two.years	1.036528	1.020840	1.064024	1.018100	0.9647595	0.9398283	0.9795852
HDL.Cholesterol.mg.dL.	1.127630	1.105769	1.154011	1.061899	0.8868154	0.8665431	0.9043483
LDL.Cholesterol.mg.dL.	1.061147	1.043063	1.086826	1.030120	0.9423764	0.9201105	0.9587150
C.Reactive.Protein.mg.L.	1.021594	1.008581	1.054341	1.010739	0.9788627	0.9484596	0.9914922
Uric.acid.mg.dL.	1.172201	1.148342	1.199897	1.082682	0.8530962	0.8334046	0.8708211

BFP was treated as a categorical variable

Model 2 : Variance Inflation Factor and Tolerance

Term	VIF	VIF_CI_low	VIF_CI_high	SE_factor	Tolerance	Tolerance_CI_low	Tolerance_CI_high
BFP_cat	1.067602	1.049014	1.093239	1.033248	0.9366790	0.9147132	0.9532764
Age	1.209270	1.183798	1.238273	1.099668	0.8269449	0.8075762	0.8447387
Education	1.160547	1.137143	1.187944	1.077287	0.8616628	0.8417906	0.8793965
Marital.status	1.117367	1.095954	1.143558	1.057056	0.8949610	0.8744634	0.9124469
Residence.place	1.066402	1.047898	1.092054	1.032668	0.9377325	0.9157054	0.9542910

BFP was treated as a categorical variable

Model 3 : Variance Inflation Factor and Tolerance

Term	VIF	VIF_CI_low	VIF_CI_high	SE_factor	Tolerance	Tolerance_CI_low	Tolerance_CI_high
BFP_cat	2.047694	1.990210	2.108515	1.430977	0.4883542	0.4742675	0.5024595
Age	1.350104	1.319091	1.384131	1.161940	0.7406839	0.7224751	0.7580980
Education	1.187631	1.163119	1.215825	1.089785	0.8420126	0.8224867	0.8597570
Marital.status	1.124674	1.102956	1.150973	1.060506	0.8891465	0.8688304	0.9066541
Residence.place	1.103966	1.083275	1.129797	1.050698	0.9058252	0.8851147	0.9231265
Drink	1.381749	1.349510	1.416962	1.175478	0.7237202	0.7057350	0.7410095
Smoke	1.798042	1.749967	1.849200	1.340911	0.5561604	0.5407745	0.5714393
Hypertension	1.162025	1.138611	1.189394	1.077973	0.8605667	0.8407641	0.8782632
Diabetes	1.063111	1.044883	1.088742	1.031073	0.9406353	0.9184912	0.9570447
Dyslipidemia	1.125549	1.103790	1.151870	1.060919	0.8884554	0.8681536	0.9059697
Stroke	1.036017	1.020399	1.063592	1.017849	0.9652354	0.9402104	0.9800088
Cancer	1.019333	1.006929	1.053944	1.009620	0.9810333	0.9488171	0.9931186
Lung.diseases	1.052017	1.034681	1.078019	1.025679	0.9505547	0.9276272	0.9664812
Heart.diseases	1.104437	1.083722	1.130278	1.050922	0.9054383	0.8847382	0.9227454
Liver.diseases	1.021982	1.008874	1.054451	1.010931	0.9784905	0.9483607	0.9912036
Kidney.diseases	1.030264	1.015497	1.059102	1.015019	0.9706247	0.9441961	0.9847390
Stomach.diseases	1.053651	1.036173	1.079575	1.026475	0.9490807	0.9262904	0.9650900
Sleep.duration	1.041221	1.024963	1.068067	1.020402	0.9604114	0.9362710	0.9756453
Life.satisfaction	1.108722	1.087787	1.134648	1.052958	0.9019397	0.8813305	0.9192975
Self.rated.health	1.227591	1.201427	1.257153	1.107967	0.8146037	0.7954484	0.8323433
Falls.in.the.past.two.years	1.037137	1.021373	1.064529	1.018399	0.9641928	0.9393825	0.9790746
HDL.Cholesterol.mg.dL.	1.128058	1.106179	1.154443	1.062101	0.8864796	0.8662182	0.9040124
LDL.Cholesterol.mg.dL.	1.058951	1.041039	1.084682	1.029053	0.9443308	0.9219295	0.9605791
C.Reactive.Protein.mg.L.	1.021899	1.008811	1.054424	1.010890	0.9785707	0.9483849	0.9912657
Uric.acid.mg.dL.	1.179826	1.155645	1.207764	1.086198	0.8475824	0.8279764	0.8653174

BMI was treated as a continuous variable

Model 2 : Variance Inflation Factor and Tolerance

Term	VIF	VIF_CI_low	VIF_CI_high	SE_factor	Tolerance	Tolerance_CI_low	Tolerance_CI_high
BMI	1.089178	1.069241	1.114854	1.043637	0.9181240	0.8969788	0.9352425
Age	1.250592	1.223433	1.281052	1.118299	0.7996215	0.7806086	0.8173722
Sex	1.093744	1.073552	1.119478	1.045822	0.9142908	0.8932731	0.9314870
Education	1.190925	1.166216	1.219308	1.091295	0.8396833	0.8201374	0.8574742
Marital.status	1.124983	1.103199	1.151364	1.060652	0.8889027	0.8685351	0.9064546
Residence.place	1.073430	1.054448	1.099028	1.036064	0.9315935	0.9098945	0.9483635

BMI was treated as a continuous variable

Model 3 : Variance Inflation Factor and Tolerance

Term	VIF	VIF_CI_low	VIF_CI_high	SE_factor	Tolerance	Tolerance_CI_low	Tolerance_CI_high
BMI	1.376834	1.344782	1.411866	1.173386	0.7263040	0.7082827	0.7436150
Age	1.416181	1.382611	1.452696	1.190034	0.7061244	0.6883754	0.7232690
Sex	2.517434	2.442287	2.596497	1.586642	0.3972299	0.3851343	0.4094524
Education	1.208151	1.182782	1.237041	1.099159	0.8277113	0.8083806	0.8454647
Marital.status	1.130107	1.108131	1.156550	1.063065	0.8848718	0.8646406	0.9024206
Residence.place	1.105134	1.084382	1.130991	1.051254	0.9048674	0.8841804	0.9221847
Drink	1.460018	1.424766	1.498195	1.208312	0.6849233	0.6674700	0.7018697
Smoke	2.035611	1.978577	2.095970	1.426748	0.4912529	0.4771061	0.5054138
Hypertension	1.179941	1.155753	1.207885	1.086251	0.8474999	0.8278931	0.8652366
Diabetes	1.065193	1.046813	1.090791	1.032082	0.9387969	0.9167662	0.9552808
Dyslipidemia	1.134112	1.111949	1.160663	1.064947	0.8817473	0.8615769	0.8993221
Stroke	1.035584	1.020023	1.063238	1.017637	0.9656384	0.9405233	0.9803696
Cancer	1.019212	1.006842	1.053946	1.009560	0.9811504	0.9488150	0.9932047
Lung.diseases	1.051803	1.034485	1.077819	1.025575	0.9507481	0.9277993	0.9666647
Heart.diseases	1.106701	1.085868	1.132589	1.051999	0.9035864	0.8829330	0.9209224
Liver.diseases	1.021681	1.008647	1.054362	1.010782	0.9787795	0.9484410	0.9914275
Kidney.diseases	1.030073	1.015336	1.058968	1.014925	0.9708055	0.9443155	0.9848953
Stomach.diseases	1.057392	1.039602	1.083174	1.028296	0.9457232	0.9232129	0.9619068
Sleep.duration	1.043038	1.026577	1.069694	1.021292	0.9587379	0.9348467	0.9741110
Life.satisfaction	1.109769	1.088780	1.135720	1.053456	0.9010887	0.8804987	0.9184596
Self.rated.health	1.229179	1.202948	1.258799	1.108683	0.8135513	0.7944078	0.8312909
Falls.in.the.past.two.years	1.036860	1.021130	1.064299	1.018263	0.9644501	0.9395852	0.9793068
HDL.Cholesterol.mg.dL.	1.181412	1.157162	1.209405	1.086928	0.8464447	0.8268532	0.8641835
LDL.Cholesterol.mg.dL.	1.062493	1.044309	1.088139	1.030773	0.9411826	0.9190001	0.9575707
C.Reactive.Protein.mg.L.	1.021593	1.008581	1.054336	1.010739	0.9788635	0.9484642	0.9914921
Uric.acid.mg.dL.	1.250357	1.223271	1.280729	1.118194	0.7997716	0.7808056	0.8174801

BMI was treated as a categorical variable

Model 2 : Variance Inflation Factor and Tolerance

Term	VIF	VIF_CI_low	VIF_CI_high	SE_factor	Tolerance	Tolerance_CI_low	Tolerance_CI_high
BMI_cat	1.092134	1.072035	1.117841	1.045052	0.9156383	0.8945814	0.9328051
Age	1.252216	1.224996	1.282728	1.119024	0.7985844	0.7795885	0.8163289
Sex	1.092640	1.072513	1.118354	1.045294	0.9152142	0.8941712	0.9323894
Education	1.191835	1.167092	1.220242	1.091712	0.8390424	0.8195095	0.8568308
Marital.status	1.125515	1.103710	1.151905	1.060903	0.8884821	0.8681275	0.9060350
Residence.place	1.074311	1.055276	1.099902	1.036490	0.9308289	0.9091716	0.9476197

BMI was treated as a categorical variable

Model 3 : Variance Inflation Factor and Tolerance

Term	VIF	VIF_CI_low	VIF_CI_high	SE_factor	Tolerance	Tolerance_CI_low	Tolerance_CI_high
BMI_cat	1.373874	1.341942	1.408787	1.172124	0.7278690	0.7098304	0.7451887
Age	1.410353	1.377014	1.446640	1.187583	0.7090424	0.6912569	0.7262092
Sex	2.514239	2.439225	2.593162	1.585635	0.3977347	0.3856296	0.4099662
Education	1.208735	1.183346	1.237639	1.099425	0.8273115	0.8079900	0.8450615
Marital.status	1.130686	1.108687	1.157139	1.063337	0.8844186	0.8642004	0.9019681
Residence.place	1.105865	1.085078	1.131730	1.051601	0.9042699	0.8836029	0.9215929
Drink	1.460120	1.424871	1.498293	1.208354	0.6848753	0.6674260	0.7018180
Smoke	2.036603	1.979542	2.096988	1.427096	0.4910138	0.4768745	0.5051674
Hypertension	1.177118	1.153055	1.204965	1.084951	0.8495324	0.8298998	0.8672615
Diabetes	1.064668	1.046328	1.090268	1.031828	0.9392599	0.9172056	0.9557231
Dyslipidemia	1.132860	1.110759	1.159372	1.064359	0.8827212	0.8625362	0.9002850
Stroke	1.035753	1.020171	1.063370	1.017719	0.9654813	0.9404066	0.9802274
Cancer	1.019229	1.006855	1.053936	1.009568	0.9811342	0.9488244	0.9931915
Lung.diseases	1.052166	1.034818	1.078158	1.025752	0.9504201	0.9275082	0.9663532
Heart.diseases	1.105111	1.084363	1.130961	1.051242	0.9048869	0.8842036	0.9222008
Liver.diseases	1.021918	1.008827	1.054426	1.010900	0.9785522	0.9483836	0.9912506
Kidney.diseases	1.030340	1.015562	1.059153	1.015057	0.9705535	0.9441509	0.9846769
Stomach.diseases	1.055971	1.038300	1.081797	1.027605	0.9469953	0.9243876	0.9631132
Sleep.duration	1.042639	1.026224	1.069329	1.021097	0.9591044	0.9351657	0.9744458
Life.satisfaction	1.109824	1.088835	1.135771	1.053482	0.9010442	0.8804595	0.9184126
Self.rated.health	1.231487	1.205168	1.261183	1.109724	0.8120265	0.7929066	0.8297601
Falls.in.the.past.two.years	1.037118	1.021358	1.064510	1.018390	0.9642101	0.9393994	0.9790891
HDL.Cholesterol.mg.dL.	1.184539	1.160160	1.212628	1.088365	0.8442104	0.8246550	0.8619499
LDL.Cholesterol.mg.dL.	1.064099	1.045801	1.089709	1.031552	0.9397619	0.9176762	0.9562053
C.Reactive.Protein.mg.L.	1.022199	1.009039	1.054519	1.011038	0.9782835	0.9483000	0.9910423
Uric.acid.mg.dL.	1.247834	1.220855	1.278109	1.117065	0.8013887	0.7824058	0.8190984

WHtR was analyzed as a standardized continuous variable

Model 2 : Variance Inflation Factor and Tolerance

Term	VIF	VIF_CI_low	VIF_CI_high	SE_factor	Tolerance	Tolerance_CI_low	Tolerance_CI_high
WHtR_normalized	1.151374	1.128374	1.178494	1.073021	0.8685277	0.8485408	0.8862306
Age	1.241433	1.214644	1.271566	1.114196	0.8055205	0.7864316	0.8232865
Sex	1.203066	1.177848	1.231859	1.096843	0.8312099	0.8117815	0.8490059
Education	1.187709	1.163135	1.215985	1.089821	0.8419570	0.8223788	0.8597452
Marital.status	1.123893	1.102162	1.150247	1.060138	0.8897643	0.8693786	0.9073077
Residence.place	1.067431	1.048854	1.093073	1.033166	0.9368284	0.9148516	0.9534217

WHtR was analyzed as a standardized continuous variable

Model 3 : Variance Inflation Factor and Tolerance

Term	VIF	VIF_CI_low	VIF_CI_high	SE_factor	Tolerance	Tolerance_CI_low	Tolerance_CI_high
WHtR_normalized	1.414138	1.380647	1.450576	1.189175	0.7071445	0.6893814	0.7242980
Age	1.352157	1.321062	1.386265	1.162823	0.7395589	0.7213630	0.7569669
Sex	2.651668	2.571480	2.735948	1.628394	0.3771211	0.3655040	0.3888812
Education	1.207185	1.181856	1.236042	1.098720	0.8283735	0.8090339	0.8461270
Marital.status	1.129214	1.107280	1.155633	1.062645	0.8855715	0.8653263	0.9031140
Residence.place	1.102286	1.081681	1.128088	1.049898	0.9072058	0.8864554	0.9244871
Drink	1.460952	1.425664	1.499164	1.208698	0.6844854	0.6670383	0.7014275
Smoke	2.031447	1.974570	2.091644	1.425289	0.4922598	0.4780927	0.5064394
Hypertension	1.174331	1.150383	1.202094	1.083666	0.8515484	0.8318819	0.8692757
Diabetes	1.067356	1.048823	1.092924	1.033129	0.9368948	0.9149767	0.9534501
Dyslipidemia	1.124984	1.103250	1.151294	1.060653	0.8889012	0.8685881	0.9064128
Stroke	1.035456	1.019913	1.063132	1.017574	0.9657581	0.9406166	0.9804762
Cancer	1.019252	1.006870	1.053947	1.009580	0.9811119	0.9488143	0.9931766
Lung.diseases	1.051033	1.033783	1.077090	1.025199	0.9514452	0.9284279	0.9673209
Heart.diseases	1.102947	1.082307	1.128761	1.050213	0.9066623	0.8859269	0.9239521
Liver.diseases	1.021731	1.008685	1.054377	1.010807	0.9787312	0.9484274	0.9913903
Kidney.diseases	1.030023	1.015295	1.058932	1.014900	0.9708524	0.9443473	0.9849356
Stomach.diseases	1.056495	1.038777	1.082307	1.027859	0.9465263	0.9239519	0.9626704
Sleep.duration	1.042727	1.026300	1.069414	1.021140	0.9590241	0.9350915	0.9743742
Life.satisfaction	1.110426	1.089404	1.136392	1.053768	0.9005550	0.8799782	0.9179330
Self.rated.health	1.227174	1.201025	1.256724	1.107779	0.8148806	0.7957198	0.8326223
Falls.in.the.past.two.years	1.038652	1.022696	1.065824	1.019143	0.9627866	0.9382409	0.9778076
HDL.Cholesterol.mg.dL.	1.173696	1.149775	1.201438	1.083373	0.8520092	0.8323359	0.8697354
LDL.Cholesterol.mg.dL.	1.062950	1.044732	1.088587	1.030995	0.9407783	0.9186224	0.9571831
C.Reactive.Protein.mg.L.	1.021766	1.008711	1.054388	1.010825	0.9786974	0.9484177	0.9913643
Uric.acid.mg.dL.	1.251075	1.223960	1.281472	1.118514	0.7993128	0.7803527	0.8170201

WHtR was treated as a categorical variable

Model 2 : Variance Inflation Factor and Tolerance

Term	VIF	VIF_CI_low	VIF_CI_high	SE_factor	Tolerance	Tolerance_CI_low	Tolerance_CI_high
WHtR_cat	1.137833	1.115452	1.164554	1.066693	0.8788633	0.8586977	0.8964979
Age	1.240315	1.213576	1.270402	1.113694	0.8062468	0.7871524	0.8240112
Sex	1.187278	1.162727	1.215533	1.089623	0.8422626	0.8226841	0.8600472
Education	1.188447	1.163846	1.216741	1.090159	0.8414342	0.8218674	0.8592199
Marital.status	1.124438	1.102685	1.150800	1.060395	0.8893331	0.8689607	0.9068777
Residence.place	1.066620	1.048102	1.092266	1.032773	0.9375413	0.9155279	0.9541056

WHtR was treated as a categorical variable

Model 3 : Variance Inflation Factor and Tolerance

Term	VIF	VIF_CI_low	VIF_CI_high	SE_factor	Tolerance	Tolerance_CI_low	Tolerance_CI_high
WHtR_cat	1.382403	1.350142	1.417637	1.175756	0.7233781	0.7053992	0.7406631
Age	1.353282	1.322148	1.387424	1.163306	0.7389445	0.7207602	0.7563449
Sex	2.631103	2.551702	2.714568	1.622068	0.3800687	0.3683828	0.3918953
Education	1.207698	1.182352	1.236567	1.098953	0.8280213	0.8086904	0.8457715
Marital.status	1.129750	1.107794	1.156177	1.062897	0.8851519	0.8649192	0.9026950
Residence.place	1.102086	1.081495	1.127879	1.049803	0.9073701	0.8866195	0.9246457
Drink	1.461005	1.425722	1.499213	1.208721	0.6844602	0.6670168	0.7013988
Smoke	2.030009	1.973196	2.090138	1.424784	0.4926087	0.4784373	0.5067920
Hypertension	1.171008	1.147206	1.198657	1.082131	0.8539655	0.8342668	0.8716830
Diabetes	1.065677	1.047265	1.091262	1.032317	0.9383702	0.9163703	0.9548678
Dyslipidemia	1.124397	1.102695	1.150686	1.060376	0.8893652	0.8690466	0.9068691
Stroke	1.035568	1.020012	1.063218	1.017629	0.9656532	0.9405410	0.9803807
Cancer	1.019350	1.006942	1.053939	1.009629	0.9810174	0.9488213	0.9931063
Lung.diseases	1.051388	1.034109	1.077420	1.025372	0.9511235	0.9281433	0.9670157
Heart.diseases	1.103010	1.082370	1.128820	1.050243	0.9066105	0.8858808	0.9238981
Liver.diseases	1.021820	1.008753	1.054395	1.010851	0.9786459	0.9484114	0.9913230
Kidney.diseases	1.030319	1.015544	1.059138	1.015046	0.9705730	0.9441642	0.9846936
Stomach.diseases	1.056423	1.038714	1.082233	1.027824	0.9465904	0.9240158	0.9627286
Sleep.duration	1.042785	1.026354	1.069460	1.021168	0.9589709	0.9350517	0.9743230
Life.satisfaction	1.110855	1.089815	1.136824	1.053971	0.9002075	0.8796435	0.9175871
Self.rated.health	1.227899	1.201725	1.257468	1.108106	0.8143994	0.7952486	0.8321370
Falls.in.the.past.two.years	1.038555	1.022614	1.065734	1.019095	0.9628762	0.9383200	0.9778864
HDL.Cholesterol.mg.dL.	1.173334	1.149432	1.201058	1.083205	0.8522723	0.8325990	0.8699945
LDL.Cholesterol.mg.dL.	1.061706	1.043584	1.087363	1.030391	0.9418805	0.9196561	0.9582365
C.Reactive.Protein.mg.L.	1.021952	1.008852	1.054436	1.010916	0.9785198	0.9483739	0.9912255
Uric.acid.mg.dL.	1.248575	1.221566	1.278877	1.117397	0.8009128	0.7819360	0.8186213

TyG was treated as a continuous variable

Model 2 : Variance Inflation Factor and Tolerance

Term	VIF	VIF_CI_low	VIF_CI_high	SE_factor	Tolerance	Tolerance_CI_low	Tolerance_CI_high
TyG	1.015330	1.004231	1.055548	1.007636	0.9849017	0.9473754	0.9957872
Age	1.225279	1.199146	1.254841	1.106923	0.8161405	0.7969135	0.8339265
Sex	1.078069	1.058792	1.103666	1.038301	0.9275843	0.9060709	0.9444723
Education	1.187440	1.162878	1.215707	1.089697	0.8421475	0.8225666	0.8599354
Marital.status	1.123779	1.102053	1.150130	1.060085	0.8898545	0.8694669	0.9073970
Residence.place	1.060289	1.042234	1.086062	1.029703	0.9431393	0.9207577	0.9594775

TyG was treated as a continuous variable

Model 3 : Variance Inflation Factor and Tolerance

Term	VIF	VIF_CI_low	VIF_CI_high	SE_factor	Tolerance	Tolerance_CI_low	Tolerance_CI_high
TyG	1.612633	1.571568	1.656648	1.269895	0.6201040	0.6036286	0.6363073
Age	1.350683	1.319645	1.384735	1.162189	0.7403663	0.7221598	0.7577798
Sex	2.543368	2.467247	2.623439	1.594794	0.3931794	0.3811791	0.4053101
Education	1.207232	1.181900	1.236090	1.098741	0.8283415	0.8090024	0.8460950
Marital.status	1.128693	1.106783	1.155098	1.062400	0.8859807	0.8657273	0.9035196
Residence.place	1.099479	1.079022	1.125231	1.048560	0.9095219	0.8887061	0.9267653
Drink	1.460873	1.425588	1.499083	1.208666	0.6845223	0.6670746	0.7014647
Smoke	2.023130	1.966565	2.083004	1.422368	0.4942837	0.4800758	0.5085008
Hypertension	1.141496	1.118993	1.168253	1.068408	0.8760437	0.8559790	0.8936603
Diabetes	1.220821	1.194932	1.250150	1.104908	0.8191207	0.7999043	0.8368680
Dyslipidemia	1.120441	1.098925	1.146637	1.058509	0.8925057	0.8721156	0.9099803
Stroke	1.035592	1.020030	1.063244	1.017640	0.9656316	0.9405179	0.9803635
Cancer	1.019325	1.006923	1.053949	1.009616	0.9810410	0.9488125	0.9931249
Lung.diseases	1.051280	1.034008	1.077323	1.025319	0.9512215	0.9282264	0.9671104
Heart.diseases	1.102073	1.081479	1.127871	1.049796	0.9073813	0.8866261	0.9246598
Liver.diseases	1.022239	1.009068	1.054542	1.011059	0.9782444	0.9482789	0.9910134
Kidney.diseases	1.030092	1.015353	1.058982	1.014934	0.9707871	0.9443031	0.9848795
Stomach.diseases	1.052542	1.035158	1.078520	1.025934	0.9500812	0.9271962	0.9660361
Sleep.duration	1.041855	1.025525	1.068634	1.020713	0.9598262	0.9357739	0.9751106
Life.satisfaction	1.107741	1.086855	1.133650	1.052493	0.9027381	0.8821065	0.9200863
Self.rated.health	1.227300	1.201146	1.256855	1.107836	0.8147964	0.7956367	0.8325380
Falls.in.the.past.two.years	1.036289	1.020634	1.063822	1.017983	0.9649814	0.9400070	0.9797828
HDL.Cholesterol.mg.dL.	1.392076	1.359435	1.427681	1.179863	0.7183516	0.7004366	0.7355997
LDL.Cholesterol.mg.dL.	1.048042	1.031071	1.074283	1.023739	0.9541605	0.9308538	0.9698656
C.Reactive.Protein.mg.L.	1.023240	1.009833	1.054927	1.011553	0.9772882	0.9479332	0.9902630
Uric.acid.mg.dL.	1.265870	1.238164	1.296799	1.125109	0.7899707	0.7711296	0.8076477

TyG was treated as a categorical variable

Model 2 : Variance Inflation Factor and Tolerance

Term	VIF	VIF_CI_low	VIF_CI_high	SE_factor	Tolerance	Tolerance_CI_low	Tolerance_CI_high
TyG_cat	1.022267	1.009070	1.054664	1.011072	0.9782180	0.9481689	0.9910112
Age	1.226954	1.200757	1.256568	1.107679	0.8150268	0.7958183	0.8328079
Sex	1.082890	1.063323	1.108503	1.040620	0.9234549	0.9021174	0.9404480
Education	1.187724	1.163154	1.215994	1.089827	0.8419467	0.8223726	0.8597316
Marital.status	1.123463	1.101756	1.149800	1.059935	0.8901049	0.8697162	0.9076418
Residence.place	1.061735	1.043573	1.087467	1.030405	0.9418546	0.9195678	0.9582464

TyG was treated as a categorical variable

Model 3 : Variance Inflation Factor and Tolerance

Term	VIF	VIF_CI_low	VIF_CI_high	SE_factor	Tolerance	Tolerance_CI_low	Tolerance_CI_high
TyG_cat	1.552687	1.513906	1.594393	1.246068	0.6440450	0.6271978	0.6605429
Age	1.352293	1.321198	1.386399	1.162882	0.7394844	0.7212931	0.7568886
Sex	2.540809	2.464798	2.620765	1.593992	0.3935754	0.3815680	0.4057128
Education	1.207670	1.182325	1.236538	1.098940	0.8280408	0.8087096	0.8457910
Marital.status	1.128486	1.106590	1.154880	1.062302	0.8861431	0.8658906	0.9036775
Residence.place	1.099837	1.079365	1.125590	1.048731	0.9092255	0.8884226	0.9264708
Drink	1.460699	1.425428	1.498894	1.208594	0.6846038	0.6671584	0.7015438
Smoke	2.023443	1.966877	2.083318	1.422478	0.4942071	0.4800035	0.5084201
Hypertension	1.142161	1.119633	1.168932	1.068719	0.8755333	0.8554819	0.8931502
Diabetes	1.162350	1.138924	1.189727	1.078124	0.8603257	0.8405289	0.8780216
Dyslipidemia	1.119131	1.097683	1.145290	1.057890	0.8935501	0.8731412	0.9110102
Stroke	1.035478	1.019934	1.063143	1.017584	0.9657376	0.9406069	0.9804559
Cancer	1.019398	1.006976	1.053941	1.009653	0.9809708	0.9488196	0.9930723
Lung.diseases	1.051360	1.034084	1.077393	1.025359	0.9511488	0.9281661	0.9670395
Heart.diseases	1.102703	1.082080	1.128508	1.050097	0.9068624	0.8861259	0.9241461
Liver.diseases	1.022541	1.009299	1.054641	1.011208	0.9779555	0.9481900	0.9907865
Kidney.diseases	1.030201	1.015445	1.059052	1.014988	0.9706846	0.9442403	0.9847896
Stomach.diseases	1.053178	1.035742	1.079121	1.026245	0.9495070	0.9266806	0.9654916
Sleep.duration	1.042244	1.025873	1.068975	1.020904	0.9594681	0.9354755	0.9747798
Life.satisfaction	1.110460	1.089440	1.136421	1.053784	0.9005275	0.8799557	0.9179029
Self.rated.health	1.228652	1.202448	1.258248	1.108446	0.8139000	0.7947557	0.8316368
Falls.in.the.past.two.years	1.036670	1.020967	1.064133	1.018170	0.9646274	0.9397324	0.9794636
HDL.Cholesterol.mg.dL.	1.354450	1.323271	1.388636	1.163808	0.7383072	0.7201313	0.7557032
LDL.Cholesterol.mg.dL.	1.082853	1.063331	1.108393	1.040602	0.9234863	0.9022067	0.9404412
C.Reactive.Protein.mg.L.	1.023055	1.009692	1.054841	1.011462	0.9774646	0.9480102	0.9904008
Uric.acid.mg.dL.	1.261458	1.233933	1.292222	1.123147	0.7927334	0.7738609	0.8104168

TyG-WC was analyzed as a standardized continuous variable

Model 2 : Variance Inflation Factor and Tolerance

Term	VIF	VIF_CI_low	VIF_CI_high	SE_factor	Tolerance	Tolerance_CI_low	Tolerance_CI_high
TyG.WC_normalized	1.033054	1.017823	1.061299	1.016393	0.9680036	0.9422412	0.9824887
Age	1.225616	1.199469	1.255190	1.107075	0.8159164	0.7966924	0.8337021
Sex	1.077015	1.057804	1.102610	1.037793	0.9284924	0.9069387	0.9453548
Education	1.189616	1.164962	1.217955	1.090695	0.8406073	0.8210483	0.8583972
Marital.status	1.124950	1.103168	1.151330	1.060637	0.8889285	0.8685604	0.9064802
Residence.place	1.074549	1.055495	1.100145	1.036604	0.9306232	0.9089710	0.9474230

TyG-WC was analyzed as a standardized continuous variable

Model 3 : Variance Inflation Factor and Tolerance

Term	VIF	VIF_CI_low	VIF_CI_high	SE_factor	Tolerance	Tolerance_CI_low	Tolerance_CI_high
TyG.WC_normalized	1.637653	1.595639	1.682630	1.279708	0.6106299	0.5943076	0.6267080
Age	1.352959	1.321832	1.387096	1.163168	0.7391208	0.7209306	0.7565257
Sex	2.521382	2.446086	2.600598	1.587886	0.3966079	0.3845269	0.4088164
Education	1.207891	1.182532	1.236772	1.099041	0.8278894	0.8085564	0.8456429
Marital.status	1.130076	1.108101	1.156518	1.063050	0.8848964	0.8646646	0.9024449
Residence.place	1.103426	1.082762	1.129250	1.050441	0.9062681	0.8855434	0.9235640
Drink	1.463715	1.428322	1.502033	1.209841	0.6831929	0.6657642	0.7001222
Smoke	2.025774	1.969110	2.085751	1.423297	0.4936385	0.4794436	0.5078437
Hypertension	1.179252	1.155093	1.207174	1.085934	0.8479951	0.8283810	0.8657308
Diabetes	1.138621	1.116250	1.165297	1.067062	0.8782551	0.8581501	0.8958563
Dyslipidemia	1.130887	1.108875	1.157351	1.063432	0.8842613	0.8640421	0.9018153
Stroke	1.035336	1.019809	1.063034	1.017515	0.9658696	0.9407034	0.9805755
Cancer	1.019366	1.006952	1.053950	1.009637	0.9810018	0.9488113	0.9930963
Lung.diseases	1.051172	1.033910	1.077221	1.025267	0.9513193	0.9283145	0.9672024
Heart.diseases	1.104906	1.084165	1.130759	1.051145	0.9050540	0.8843620	0.9223685
Liver.diseases	1.021884	1.008800	1.054424	1.010883	0.9785843	0.9483847	0.9912769
Kidney.diseases	1.030012	1.015286	1.058925	1.014895	0.9708623	0.9443539	0.9849440
Stomach.diseases	1.056060	1.038378	1.081889	1.027648	0.9469156	0.9243094	0.9630401
Sleep.duration	1.042643	1.026225	1.069339	1.021099	0.9591014	0.9351575	0.9744454
Life.satisfaction	1.110419	1.089397	1.136384	1.053764	0.9005612	0.8799842	0.9179391
Self.rated.health	1.228405	1.202207	1.257999	1.108335	0.8140634	0.7949132	0.8318038
Falls.in.the.past.two.years	1.037163	1.021394	1.064554	1.018412	0.9641690	0.9393604	0.9790543
HDL.Cholesterol.mg.dL.	1.364414	1.332843	1.398980	1.168081	0.7329154	0.7148066	0.7502760
LDL.Cholesterol.mg.dL.	1.058112	1.040264	1.083871	1.028646	0.9450797	0.9226194	0.9612943
C.Reactive.Protein.mg.L.	1.021688	1.008652	1.054364	1.010786	0.9787722	0.9484389	0.9914218
Uric.acid.mg.dL.	1.271432	1.243505	1.302563	1.127578	0.7865147	0.7677174	0.8041787

TyG-WC was treated as a categorical variable

Model 2 : Variance Inflation Factor and Tolerance

Term	VIF	VIF_CI_low	VIF_CI_high	SE_factor	Tolerance	Tolerance_CI_low	Tolerance_CI_high
TyG.WC_cat	1.036518	1.020806	1.064096	1.018095	0.9647682	0.9397651	0.9796178
Age	1.226132	1.199969	1.255718	1.107309	0.8155727	0.7963570	0.8333546
Sex	1.080789	1.061349	1.106391	1.039610	0.9252497	0.9038400	0.9421974
Education	1.190370	1.165688	1.218728	1.091041	0.8400749	0.8205274	0.8578622
Marital.status	1.124957	1.103179	1.151333	1.060640	0.8889226	0.8685588	0.9064712
Residence.place	1.074101	1.055079	1.099693	1.036389	0.9310109	0.9093449	0.9477961

TyG-WC was treated as a categorical variable

Model 3 : Variance Inflation Factor and Tolerance

Term	VIF	VIF_CI_low	VIF_CI_high	SE_factor	Tolerance	Tolerance_CI_low	Tolerance_CI_high
TyG.WC_cat	1.567012	1.527687	1.609268	1.251804	0.6381571	0.6214005	0.6545843
Age	1.351577	1.320510	1.385656	1.162573	0.7398764	0.7216799	0.7572833
Sex	2.524760	2.449351	2.604092	1.588949	0.3960773	0.3840111	0.4082714
Education	1.208684	1.183297	1.237587	1.099402	0.8273462	0.8080243	0.8450962
Marital.status	1.130211	1.108233	1.156651	1.063114	0.8847908	0.8645653	0.9023371
Residence.place	1.103827	1.083146	1.129653	1.050632	0.9059388	0.8852274	0.9232368
Drink	1.463096	1.427733	1.501383	1.209585	0.6834822	0.6660527	0.7004111
Smoke	2.026364	1.969688	2.086352	1.423504	0.4934949	0.4793056	0.5076946
Hypertension	1.177738	1.153648	1.205605	1.085236	0.8490851	0.8294591	0.8668153
Diabetes	1.107841	1.086954	1.133747	1.052540	0.9026563	0.8820310	0.9200025
Dyslipidemia	1.126503	1.104701	1.152846	1.061369	0.8877027	0.8674185	0.9052226
Stroke	1.035650	1.020083	1.063285	1.017669	0.9655768	0.9404813	0.9803125
Cancer	1.019393	1.006972	1.053941	1.009650	0.9809762	0.9488198	0.9930762
Lung.diseases	1.051351	1.034075	1.077384	1.025354	0.9511573	0.9281738	0.9670475
Heart.diseases	1.104463	1.083748	1.130301	1.050934	0.9054177	0.8847203	0.9227236
Liver.diseases	1.022100	1.008964	1.054485	1.010990	0.9783779	0.9483300	0.9911156
Kidney.diseases	1.030093	1.015355	1.058975	1.014935	0.9707862	0.9443093	0.9848769
Stomach.diseases	1.055289	1.037674	1.081141	1.027273	0.9476076	0.9249487	0.9636941
Sleep.duration	1.042975	1.026523	1.069631	1.021261	0.9587962	0.9349023	0.9741623
Life.satisfaction	1.111372	1.090306	1.137352	1.054216	0.8997890	0.8792353	0.9171741
Self.rated.health	1.228505	1.202307	1.258096	1.108379	0.8139975	0.7948519	0.8317345
Falls.in.the.past.two.years	1.037635	1.021808	1.064948	1.018644	0.9637300	0.9390134	0.9786571
HDL.Cholesterol.mg.dL.	1.333378	1.303019	1.366779	1.154720	0.7499749	0.7316474	0.7674486
LDL.Cholesterol.mg.dL.	1.073897	1.054929	1.099415	1.036290	0.9311880	0.9095743	0.9479313
C.Reactive.Protein.mg.L.	1.022184	1.009027	1.054513	1.011031	0.9782979	0.9483046	0.9910535
Uric.acid.mg.dL.	1.269385	1.241544	1.300435	1.126670	0.7877832	0.7689736	0.8054488

TyG-BMI was analyzed as a standardized continuous variable

Model 2 : Variance Inflation Factor and Tolerance

Term	VIF	VIF_CI_low	VIF_CI_high	SE_factor	Tolerance	Tolerance_CI_low	Tolerance_CI_high
TyG.BMI_normalized	1.077298	1.058069	1.102894	1.037930	0.9282485	0.9067058	0.9451179
Age	1.240086	1.213351	1.270171	1.113592	0.8063955	0.7872954	0.8241636
Sex	1.094262	1.074042	1.120004	1.046070	0.9138578	0.8928538	0.9310622
Education	1.190163	1.165485	1.218520	1.090946	0.8402214	0.8206679	0.8580117
Marital.status	1.125419	1.103615	1.151812	1.060858	0.8885579	0.8681975	0.9061132
Residence.place	1.074841	1.055768	1.100437	1.036745	0.9303701	0.9087300	0.9471776

TyG-BMI was analyzed as a standardized continuous variable

Model 3 : Variance Inflation Factor and Tolerance

Term	VIF	VIF_CI_low	VIF_CI_high	SE_factor	Tolerance	Tolerance_CI_low	Tolerance_CI_high
TyG.BMI_normalized	1.649631	1.607163	1.695069	1.284379	0.6061963	0.5899466	0.6222144
Age	1.408563	1.375287	1.444790	1.186829	0.7099431	0.6921419	0.7271209
Sex	2.539656	2.463674	2.619583	1.593630	0.3937541	0.3817402	0.4058978
Education	1.207863	1.182506	1.236744	1.099028	0.8279084	0.8085751	0.8456618
Marital.status	1.130689	1.108685	1.157147	1.063339	0.8844166	0.8641943	0.9019692
Residence.place	1.103426	1.082762	1.129250	1.050441	0.9062683	0.8855437	0.9235643
Drink	1.462031	1.426703	1.500285	1.209145	0.6839798	0.6665399	0.7009169
Smoke	2.030188	1.973357	2.090336	1.424846	0.4925653	0.4783920	0.5067506
Hypertension	1.183444	1.159107	1.211503	1.087862	0.8449917	0.8254212	0.8627332
Diabetes	1.117326	1.095961	1.143447	1.057036	0.8949939	0.8745485	0.9124409
Dyslipidemia	1.137767	1.115435	1.164419	1.066661	0.8789148	0.8587976	0.8965112
Stroke	1.035583	1.020022	1.063237	1.017636	0.9656399	0.9405244	0.9803709
Cancer	1.019270	1.006883	1.053947	1.009589	0.9810941	0.9488140	0.9931637
Lung.diseases	1.051334	1.034058	1.077375	1.025346	0.9511724	0.9281821	0.9670642
Heart.diseases	1.105787	1.085001	1.131657	1.051564	0.9043331	0.8836602	0.9216582
Liver.diseases	1.021799	1.008736	1.054398	1.010841	0.9786660	0.9484086	0.9913400
Kidney.diseases	1.029991	1.015269	1.058910	1.014885	0.9708819	0.9443672	0.9849609
Stomach.diseases	1.056885	1.039136	1.082684	1.028049	0.9461767	0.9236304	0.9623381
Sleep.duration	1.042483	1.026083	1.069195	1.021021	0.9592484	0.9352828	0.9745804
Life.satisfaction	1.109722	1.088735	1.135672	1.053433	0.9011266	0.8805356	0.9184970
Self.rated.health	1.229183	1.202953	1.258804	1.108685	0.8135484	0.7944049	0.8312879
Falls.in.the.past.two.years	1.036708	1.020998	1.064172	1.018189	0.9645915	0.9396978	0.9794336
HDL.Cholesterol.mg.dL.	1.321777	1.291866	1.354754	1.149686	0.7565571	0.7381413	0.7740742
LDL.Cholesterol.mg.dL.	1.059711	1.041738	1.085423	1.029423	0.9436537	0.9212998	0.9599344
C.Reactive.Protein.mg.L.	1.021695	1.008657	1.054366	1.010789	0.9787658	0.9484372	0.9914170
Uric.acid.mg.dL.	1.270674	1.242776	1.301777	1.127242	0.7869840	0.7681808	0.8046499

TyG-BMI was treated as a categorical variable

Model 2 : Variance Inflation Factor and Tolerance

Term	VIF	VIF_CI_low	VIF_CI_high	SE_factor	Tolerance	Tolerance_CI_low	Tolerance_CI_high
TyG.BMI_cat	1.082353	1.062818	1.107962	1.040362	0.9239134	0.9025577	0.9408952
Age	1.241052	1.214283	1.271165	1.114025	0.8057682	0.7866799	0.8235315
Sex	1.097430	1.077041	1.123215	1.047583	0.9112195	0.8903012	0.9284694
Education	1.190721	1.166024	1.219091	1.091202	0.8398274	0.8202833	0.8576149
Marital.status	1.125268	1.103475	1.151651	1.060786	0.8886772	0.8683185	0.9062282
Residence.place	1.075211	1.056117	1.100800	1.036924	0.9300502	0.9084298	0.9468645

TyG-BMI was treated as a categorical variable

Model 3 : Variance Inflation Factor and Tolerance

Term	VIF	VIF_CI_low	VIF_CI_high	SE_factor	Tolerance	Tolerance_CI_low	Tolerance_CI_high
TyG.BMI_cat	1.597349	1.556871	1.640768	1.263863	0.6260375	0.6094707	0.6423139
Age	1.401668	1.368663	1.437627	1.183920	0.7134358	0.6955906	0.7306400
Sex	2.536956	2.461090	2.616762	1.592783	0.3941731	0.3821517	0.4063241
Education	1.208134	1.182770	1.237017	1.099151	0.8277231	0.8083961	0.8454732
Marital.status	1.130473	1.108483	1.156920	1.063237	0.8845858	0.8643643	0.9021339
Residence.place	1.104334	1.083626	1.130169	1.050873	0.9055232	0.8848230	0.9228276
Drink	1.462954	1.427596	1.501235	1.209526	0.6835486	0.6661182	0.7004782
Smoke	2.033407	1.976466	2.093668	1.425976	0.4917855	0.4776306	0.5059535
Hypertension	1.175743	1.151739	1.203546	1.084317	0.8505257	0.8308784	0.8682525
Diabetes	1.101049	1.080513	1.126824	1.049309	0.9082248	0.8874503	0.9254866
Dyslipidemia	1.130289	1.108308	1.156731	1.063151	0.8847293	0.8645050	0.9022761
Stroke	1.035612	1.020050	1.063254	1.017650	0.9656123	0.9405090	0.9803442
Cancer	1.019299	1.006905	1.053938	1.009603	0.9810666	0.9488229	0.9931423
Lung.diseases	1.052046	1.034709	1.078044	1.025693	0.9505286	0.9276063	0.9664554
Heart.diseases	1.104531	1.083813	1.130370	1.050967	0.9053618	0.8846659	0.9226686
Liver.diseases	1.021804	1.008741	1.054390	1.010843	0.9786613	0.9484159	0.9913349
Kidney.diseases	1.030140	1.015395	1.059009	1.014958	0.9707417	0.9442791	0.9848386
Stomach.diseases	1.055939	1.038270	1.081766	1.027589	0.9470241	0.9244139	0.9631405
Sleep.duration	1.042299	1.025922	1.069024	1.020931	0.9594174	0.9354324	0.9747333
Life.satisfaction	1.109765	1.088780	1.135711	1.053454	0.9010916	0.8805058	0.9184594
Self.rated.health	1.230030	1.203770	1.259675	1.109067	0.8129882	0.7938558	0.8307235
Falls.in.the.past.two.years	1.037048	1.021296	1.064451	1.018356	0.9642754	0.9394516	0.9791478
HDL.Cholesterol.mg.dL.	1.305071	1.275820	1.337424	1.142397	0.7662419	0.7477059	0.7838099
LDL.Cholesterol.mg.dL.	1.072326	1.053460	1.097849	1.035532	0.9325526	0.9108719	0.9492532
C.Reactive.Protein.mg.L.	1.022359	1.009160	1.054575	1.011118	0.9781300	0.9482495	0.9909228
Uric.acid.mg.dL.	1.265738	1.238043	1.296657	1.125050	0.7900527	0.7712142	0.8077267

TyG-WHtR was treated as a continuous variable

Model 2 : Variance Inflation Factor and Tolerance

Term	VIF	VIF_CI_low	VIF_CI_high	SE_factor	Tolerance	Tolerance_CI_low	Tolerance_CI_high
TyG.WHtR	1.111224	1.090115	1.137279	1.054146	0.8999085	0.8792919	0.9173348
Age	1.233937	1.207452	1.263804	1.110827	0.8104139	0.7912617	0.8281904
Sex	1.163617	1.140078	1.191113	1.078711	0.8593890	0.8395508	0.8771333
Education	1.187706	1.163132	1.215981	1.089819	0.8419594	0.8223812	0.8597475
Marital.status	1.124422	1.102666	1.150789	1.060388	0.8893458	0.8689689	0.9068933
Residence.place	1.069626	1.050897	1.095246	1.034227	0.9349062	0.9130367	0.9515677

TyG-WHtR was treated as a continuous variable

Model 3 : Variance Inflation Factor and Tolerance

Term	VIF	VIF_CI_low	VIF_CI_high	SE_factor	Tolerance	Tolerance_CI_low	Tolerance_CI_high
TyG.WHtR	1.713892	1.668992	1.761806	1.309157	0.5834672	0.5675993	0.5991640
Age	1.348994	1.318021	1.382983	1.161462	0.7412931	0.7230746	0.7587130
Sex	2.673806	2.592786	2.758946	1.635178	0.3739988	0.3624572	0.3856855
Education	1.207374	1.182037	1.236237	1.098806	0.8282440	0.8089062	0.8459975
Marital.status	1.129747	1.107788	1.156181	1.062896	0.8851537	0.8649168	0.9026999
Residence.place	1.100885	1.080354	1.126663	1.049231	0.9083598	0.8875772	0.9256225
Drink	1.463357	1.427977	1.501661	1.209693	0.6833603	0.6659292	0.7002912
Smoke	2.026537	1.969844	2.086544	1.423565	0.4934526	0.4792615	0.5076544
Hypertension	1.176593	1.152548	1.204428	1.084709	0.8499116	0.8302694	0.8676430
Diabetes	1.139003	1.116615	1.165690	1.067241	0.8779608	0.8578613	0.8955642
Dyslipidemia	1.128045	1.106166	1.154434	1.062095	0.8864893	0.8662257	0.9040236
Stroke	1.035274	1.019755	1.062983	1.017484	0.9659282	0.9407489	0.9806276
Cancer	1.019324	1.006921	1.053949	1.009616	0.9810427	0.9488126	0.9931262
Lung.diseases	1.051053	1.033801	1.077109	1.025209	0.9514270	0.9284114	0.9673037
Heart.diseases	1.102731	1.082103	1.128542	1.050110	0.9068392	0.8860990	0.9241263
Liver.diseases	1.021878	1.008795	1.054422	1.010880	0.9785908	0.9483866	0.9912819
Kidney.diseases	1.029946	1.015231	1.058878	1.014863	0.9709244	0.9443958	0.9849973
Stomach.diseases	1.055805	1.038144	1.081643	1.027524	0.9471447	0.9245195	0.9632576
Sleep.duration	1.042188	1.025820	1.068931	1.020876	0.9595198	0.9355139	0.9748296
Life.satisfaction	1.110242	1.089229	1.136203	1.053680	0.9007046	0.8801241	0.9180806
Self.rated.health	1.227535	1.201371	1.257098	1.107942	0.8146409	0.7954832	0.8323822
Falls.in.the.past.two.years	1.037749	1.021906	1.065051	1.018700	0.9636240	0.9389218	0.9785639
HDL.Cholesterol.mg.dL.	1.338358	1.307799	1.371951	1.156874	0.7471842	0.7288892	0.7646433
LDL.Cholesterol.mg.dL.	1.059051	1.041130	1.084782	1.029102	0.9442413	0.9218442	0.9604951
C.Reactive.Protein.mg.L.	1.021631	1.008610	1.054347	1.010758	0.9788267	0.9484541	0.9914638
Uric.acid.mg.dL.	1.273968	1.245940	1.305190	1.128702	0.7849491	0.7661718	0.8026071

TyG-WHtR was treated as a categorical variable

Model 2 : Variance Inflation Factor and Tolerance

Term	VIF	VIF_CI_low	VIF_CI_high	SE_factor	Tolerance	Tolerance_CI_low	Tolerance_CI_high
TyG.WHtR_cat	1.107280	1.086374	1.133246	1.052274	0.9031137	0.8824208	0.9204930
Age	1.233097	1.206651	1.262928	1.110449	0.8109661	0.7918105	0.8287404
Sex	1.158371	1.135066	1.185698	1.076277	0.8632810	0.8433849	0.8810063
Education	1.188431	1.163831	1.216725	1.090152	0.8414455	0.8218786	0.8592311
Marital.status	1.124644	1.102881	1.151012	1.060492	0.8891700	0.8688010	0.9067162
Residence.place	1.070400	1.051622	1.096009	1.034602	0.9342298	0.9124013	0.9509116

TyG-WHtR was treated as a categorical variable

Model 3 : Variance Inflation Factor and Tolerance

Term	VIF	VIF_CI_low	VIF_CI_high	SE_factor	Tolerance	Tolerance_CI_low	Tolerance_CI_high
TyG.WHtR_cat	1.616961	1.575739	1.661133	1.271598	0.6184442	0.6019986	0.6346228
Age	1.348779	1.317821	1.382753	1.161370	0.7414112	0.7231947	0.7588286
Sex	2.645153	2.565224	2.729163	1.626392	0.3780500	0.3664127	0.3898295
Education	1.207393	1.182059	1.236251	1.098814	0.8282311	0.8088973	0.8459813
Marital.status	1.129757	1.107801	1.156185	1.062900	0.8851461	0.8649135	0.9026892
Residence.place	1.102048	1.081460	1.127841	1.049785	0.9074011	0.8866497	0.9246762
Drink	1.462735	1.427386	1.501008	1.209436	0.6836508	0.6662190	0.7005814
Smoke	2.026522	1.969840	2.086516	1.423560	0.4934563	0.4792678	0.5076554
Hypertension	1.172031	1.148186	1.199714	1.082604	0.8532195	0.8335320	0.8709391
Diabetes	1.109080	1.088129	1.135011	1.053129	0.9016483	0.8810486	0.9190085
Dyslipidemia	1.123642	1.101976	1.149912	1.060020	0.8899633	0.8696320	0.9074612
Stroke	1.035787	1.020201	1.063398	1.017736	0.9654499	0.9403819	0.9801993
Cancer	1.019622	1.007136	1.053954	1.009763	0.9807558	0.9488079	0.9929146
Lung.diseases	1.051483	1.034196	1.077510	1.025418	0.9510377	0.9280660	0.9669349
Heart.diseases	1.102697	1.082074	1.128501	1.050094	0.9068676	0.8861309	0.9241512
Liver.diseases	1.021993	1.008883	1.054450	1.010937	0.9784802	0.9483618	0.9911949
Kidney.diseases	1.030164	1.015415	1.059026	1.014970	0.9707189	0.9442636	0.9848191
Stomach.diseases	1.054695	1.037130	1.080571	1.026984	0.9481412	0.9254365	0.9641997
Sleep.duration	1.042528	1.026125	1.069229	1.021043	0.9592067	0.9352530	0.9745398
Life.satisfaction	1.110011	1.089013	1.135962	1.053571	0.9008920	0.8803111	0.9182625
Self.rated.health	1.227607	1.201445	1.257166	1.107974	0.8145931	0.7954398	0.8323310
Falls.in.the.past.two.years	1.037454	1.021651	1.064794	1.018555	0.9638978	0.9391486	0.9788082
HDL.Cholesterol.mg.dL.	1.309813	1.280376	1.342341	1.144471	0.7634676	0.7449670	0.7810207
LDL.Cholesterol.mg.dL.	1.072884	1.053981	1.098405	1.035801	0.9320673	0.9104108	0.9487833
C.Reactive.Protein.mg.L.	1.021690	1.008655	1.054355	1.010787	0.9787706	0.9484471	0.9914191
Uric.acid.mg.dL.	1.266291	1.238573	1.297229	1.125296	0.7897082	0.7708741	0.8073809
