# Supplementary material for: Association between obesity indices, insulin resistance markers, and osteoarthritis in middle-aged and elderly Chinese adults
Source: Front Nutr. 2025 Oct 31;12:1627421. doi: 10.3389/fnut.2025.1627421 (PMC12617301; doi:10.3389/fnut.2025.1627421)
Supplement: SUPPLEMENTARY TABLE S2 — Baseline characteristics of multivariate cox regression analysis. [file Table_2.docx]

| Characteristic | OA | | | p-value |
| --- | --- | --- | --- | --- |
|  | **Overall**  **N = 5,718** | **No**  **N = 3,891** | **Yes**  **N = 1,827** |  |
| Age, Median (Q1, Q3) | 58 (51, 65) | 58 (51, 65) | 58 (52, 65) | 0.503^1^ |
| Sex, n (%) |  |  |  | <0.001^2^ |
| female | 2,876 (50.3%) | 1,862 (47.9%) | 1,014 (55.5%) |  |
| male | 2,842 (49.7%) | 2,029 (52.1%) | 813 (44.5%) |  |
| Education, n (%) |  |  |  | <0.001^2^ |
| Primary school and blow | 3,827 (67.0%) | 2,463 (63.3%) | 1,364 (74.7%) |  |
| Junior high school and above | 1,887 (33.0%) | 1,425 (36.7%) | 462 (25.3%) |  |
| Marital status, n (%) |  |  |  | 0.079^2^ |
| Not married | 664 (11.6%) | 432 (11.1%) | 232 (12.7%) |  |
| Married | 5,054 (88.4%) | 3,459 (88.9%) | 1,595 (87.3%) |  |
| Residence place, n (%) |  |  |  | <0.001^2^ |
| Rural | 3,675 (64.3%) | 2,396 (61.6%) | 1,279 (70.0%) |  |
| Urban | 2,043 (35.7%) | 1,495 (38.4%) | 548 (30.0%) |  |
| Drink, n (%) |  |  |  | 0.053^2^ |
| No | 3,445 (60.3%) | 2,312 (59.4%) | 1,133 (62.1%) |  |
| Yes | 2,269 (39.7%) | 1,578 (40.6%) | 691 (37.9%) |  |
| Smoke, n (%) |  |  |  | 0.016^2^ |
| No | 3,358 (58.7%) | 2,243 (57.7%) | 1,115 (61.0%) |  |
| Yes | 2,359 (41.3%) | 1,647 (42.3%) | 712 (39.0%) |  |
| Hypertension, n (%) |  |  |  | 0.344^2^ |
| No | 2,991 (52.4%) | 2,020 (52.0%) | 971 (53.3%) |  |
| Yes | 2,716 (47.6%) | 1,866 (48.0%) | 850 (46.7%) |  |
| Diabetes, n (%) |  |  |  | 0.076^2^ |
| No | 4,866 (85.5%) | 3,291 (85.0%) | 1,575 (86.7%) |  |
| Yes | 824 (14.5%) | 583 (15.0%) | 241 (13.3%) |  |
| Dyslipidemia, n (%) |  |  |  | 0.738^2^ |
| No | 5,135 (91.1%) | 3,498 (91.0%) | 1,637 (91.2%) |  |
| Yes | 504 (8.9%) | 347 (9.0%) | 157 (8.8%) |  |
| Stroke, n (%) |  |  |  | 0.155^2^ |
| No | 5,586 (98.0%) | 3,794 (97.8%) | 1,792 (98.4%) |  |
| Yes | 116 (2.0%) | 86 (2.2%) | 30 (1.6%) |  |
| Cancer, n (%) |  |  |  | 0.841^2^ |
| No | 5,652 (99.2%) | 3,847 (99.2%) | 1,805 (99.2%) |  |
| Yes | 45 (0.8%) | 30 (0.8%) | 15 (0.8%) |  |
| Lung diseases, n (%) |  |  |  | 0.039^2^ |
| No | 5,241 (91.9%) | 3,587 (92.4%) | 1,654 (90.8%) |  |
| Yes | 461 (8.1%) | 294 (7.6%) | 167 (9.2%) |  |
| Heart diseases, n (%) |  |  |  | 0.693^2^ |
| No | 5,167 (90.8%) | 3,521 (90.9%) | 1,646 (90.5%) |  |
| Yes | 526 (9.2%) | 354 (9.1%) | 172 (9.5%) |  |
| Liver diseases, n (%) |  |  |  | 0.005^2^ |
| No | 5,542 (97.4%) | 3,790 (97.8%) | 1,752 (96.6%) |  |
| Yes | 146 (2.6%) | 84 (2.2%) | 62 (3.4%) |  |
| Kidney diseases, n (%) |  |  |  | <0.001^2^ |
| No | 5,434 (95.5%) | 3,741 (96.5%) | 1,693 (93.4%) |  |
| Yes | 255 (4.5%) | 135 (3.5%) | 120 (6.6%) |  |
| Stomach diseases, n (%) |  |  |  | <0.001^2^ |
| No | 4,695 (82.3%) | 3,288 (84.6%) | 1,407 (77.4%) |  |
| Yes | 1,009 (17.7%) | 598 (15.4%) | 411 (22.6%) |  |
| Life satisfaction, n (%) |  |  |  | <0.001^2^ |
| Not at all satisfied | 81 (1.6%) | 47 (1.3%) | 34 (2.1%) |  |
| Not very satisfied | 631 (12.1%) | 379 (10.7%) | 252 (15.3%) |  |
| Somewhat satisfied | 3,219 (61.9%) | 2,221 (62.6%) | 998 (60.4%) |  |
| Very satisfied | 1,159 (22.3%) | 823 (23.2%) | 336 (20.3%) |  |
| Completely satisfied | 110 (2.1%) | 78 (2.2%) | 32 (1.9%) |  |
| Self-rated health, n (%) |  |  |  | <0.001^2^ |
| Poor | 405 (7.1%) | 309 (7.9%) | 96 (5.3%) |  |
| Fair | 1,083 (19.0%) | 816 (21.0%) | 267 (14.6%) |  |
| Good | 2,948 (51.6%) | 2,001 (51.5%) | 947 (51.9%) |  |
| Very good | 1,099 (19.2%) | 667 (17.2%) | 432 (23.7%) |  |
| Excellent | 180 (3.1%) | 96 (2.5%) | 84 (4.6%) |  |
| Falls in the past two years, n (%) |  |  |  | <0.001^2^ |
| No | 4,867 (85.8%) | 3,355 (87.0%) | 1,512 (83.3%) |  |
| Yes | 807 (14.2%) | 503 (13.0%) | 304 (16.7%) |  |
| Sleep duration, Median (Q1, Q3) | 7.00 (5.00, 8.00) | 7.00 (6.00, 8.00) | 6.00 (5.00, 8.00) | <0.001^1^ |
| HDL Cholesterol(mg/dL), Median (Q1, Q3) | 49 (40, 60) | 49 (40, 60) | 50 (41, 60) | 0.034^1^ |
| LDL Cholesterol(mg/dL), Median (Q1, Q3) | 114 (93, 137) | 114 (93, 137) | 114 (94, 137) | 0.519^1^ |
| C-Reactive Protein(mg/L), Median (Q1, Q3) | 1.01 (0.54, 2.10) | 1.00 (0.54, 2.07) | 1.04 (0.54, 2.13) | 0.555^1^ |
| Uric acid(mg/dL), Median (Q1, Q3) | 4.31 (3.59, 5.20) | 4.35 (3.61, 5.22) | 4.24 (3.55, 5.15) | 0.012^1^ |

Continuous data are shown as mean median (quartile) . Categorical data are shown as n (%).

Abbreviations: HDL Cholesterol, high-density lipoprotein cholesterol (mg/dL); LDL Cholesterol, low-density lipoprotein cholesterol (mg/dL).

^1^Wilcoxon rank sum test；^2^Pearson‘s Chi-squared test.
